# Supplementary material for: Efficient Synthesis of Fluorescent Coumarins and Phosphorous-Containing Coumarin-Type Heterocycles via Palladium Catalyzed Cross-Coupling Reactions
Source: Molecules. 2022 Nov 7;27(21):7649. doi: 10.3390/molecules27217649 (PMC9654183; doi:10.3390/molecules27217649)
Supplement: Supplementary file 1 [file molecules-27-07649-s001.zip › Supplementary_Materials_revised.pdf]

# Supplementary Materials

# Efficient synthesis of fluorescent coumarins and phosphorous containing coumarin type heterocycles via palladium cross-coupling reactions.

List of content:


1. NMR spectra of compounds **3a-3e** S2-S11
2. NMR spectra of compounds **9a-9e** S12-S17
3. NMR spectra of compounds **10a-10e** S18-S23
4. NMR spectra of compounds **11a-11d** S24-S31
5. NMR spectra of compounds **12a**, **12b**, **12d** S32-S36

$^{1}H$, $^{13}C$ and $^{31}P$ NMR spectra of compound **3a**

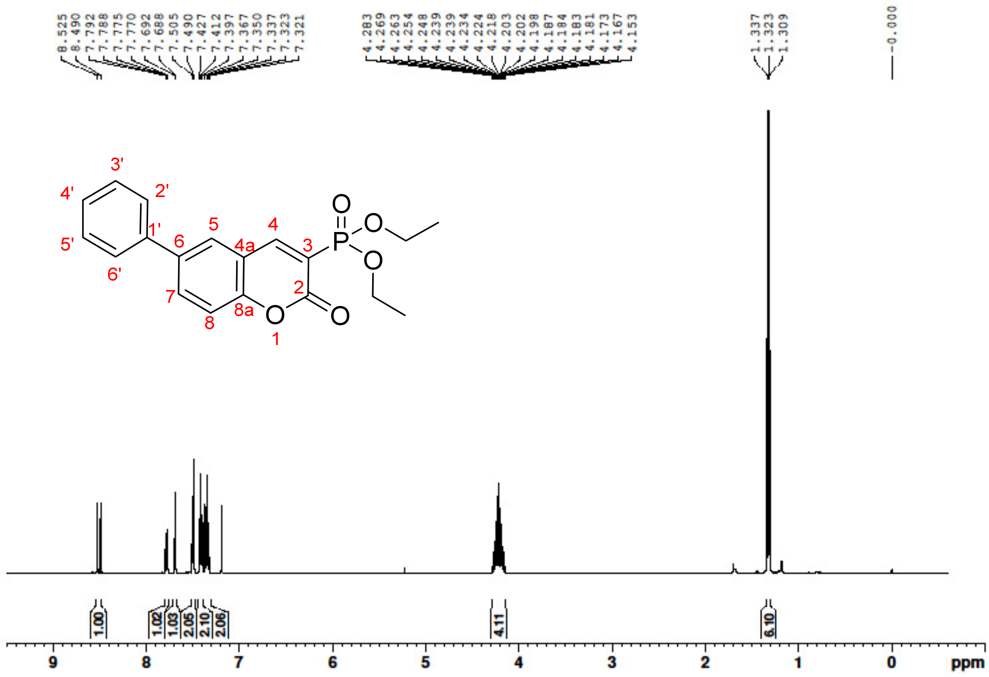

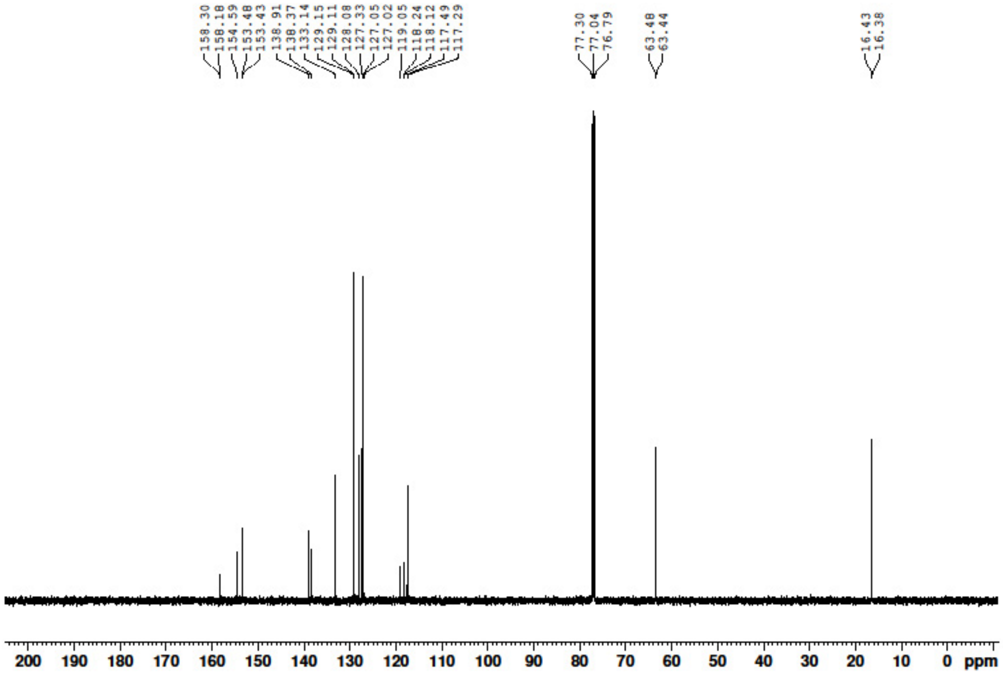

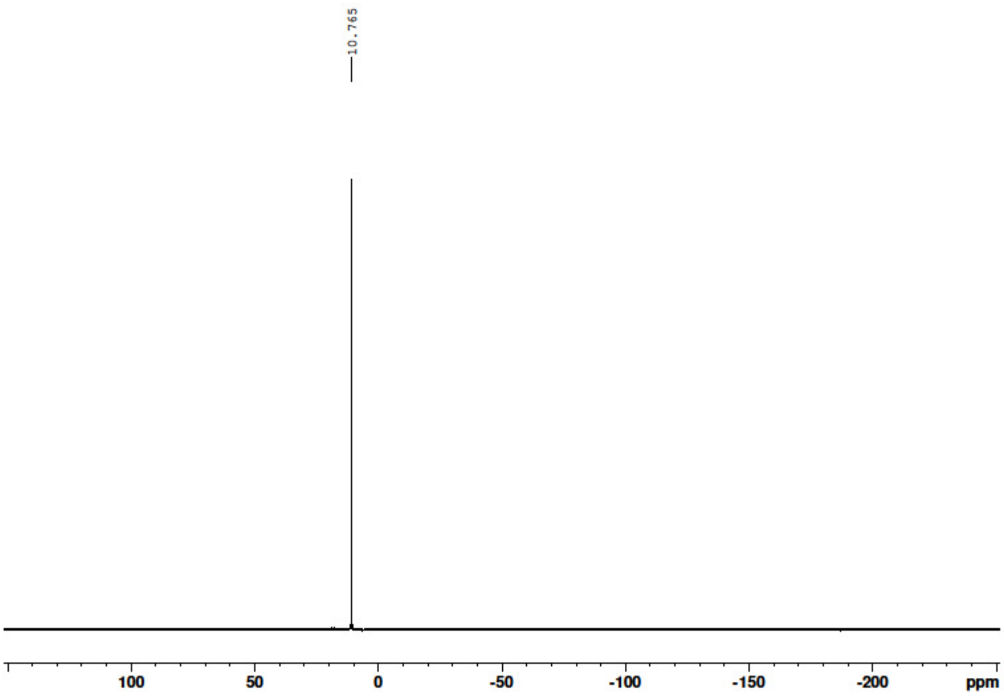

10.765
100
50
0
-50
-100
-150
-200
ppm

$^{1}H$, $^{13}C$ and $^{31}P$ NMR spectra of compound **3b**

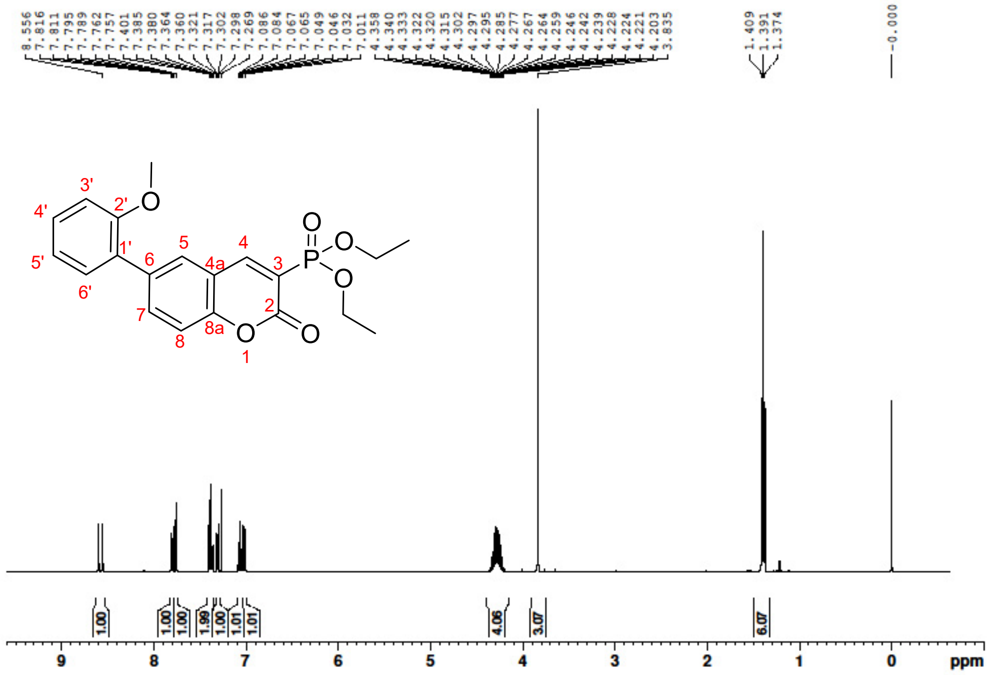


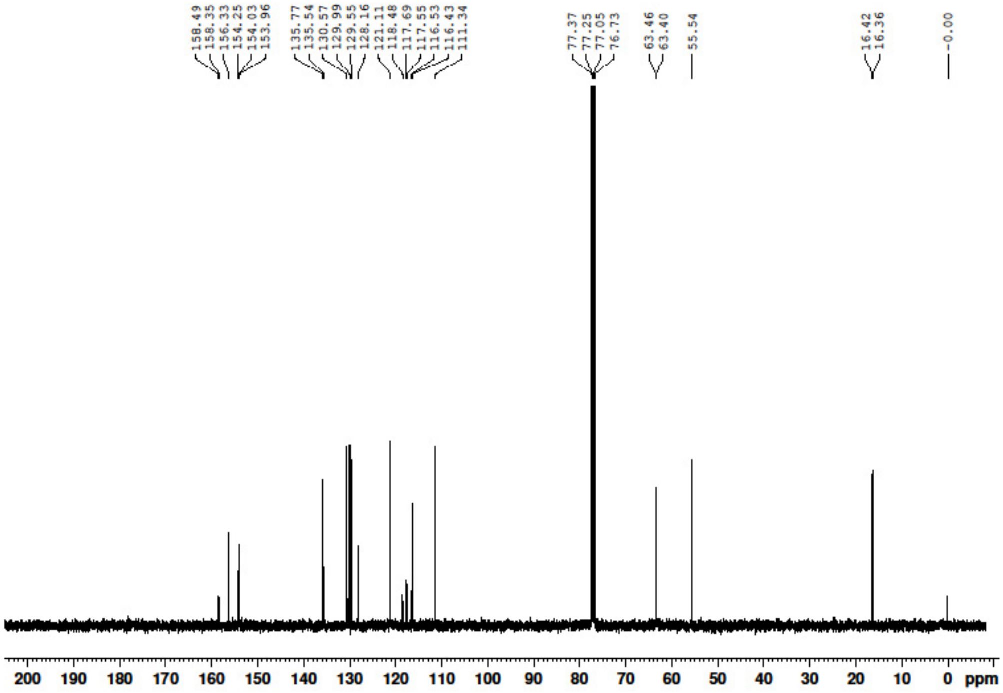

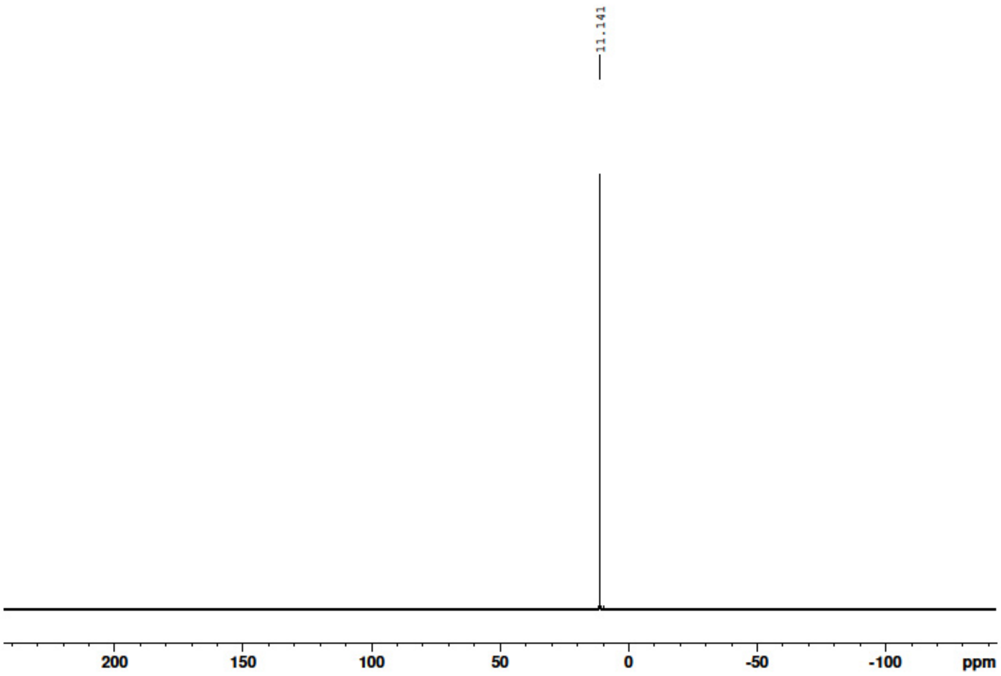
11.141
200
150
100
50
0
-50
-100
ppm

$^{1}H$, $^{13}C$ and $^{31}P$ NMR spectra of compound **3c**

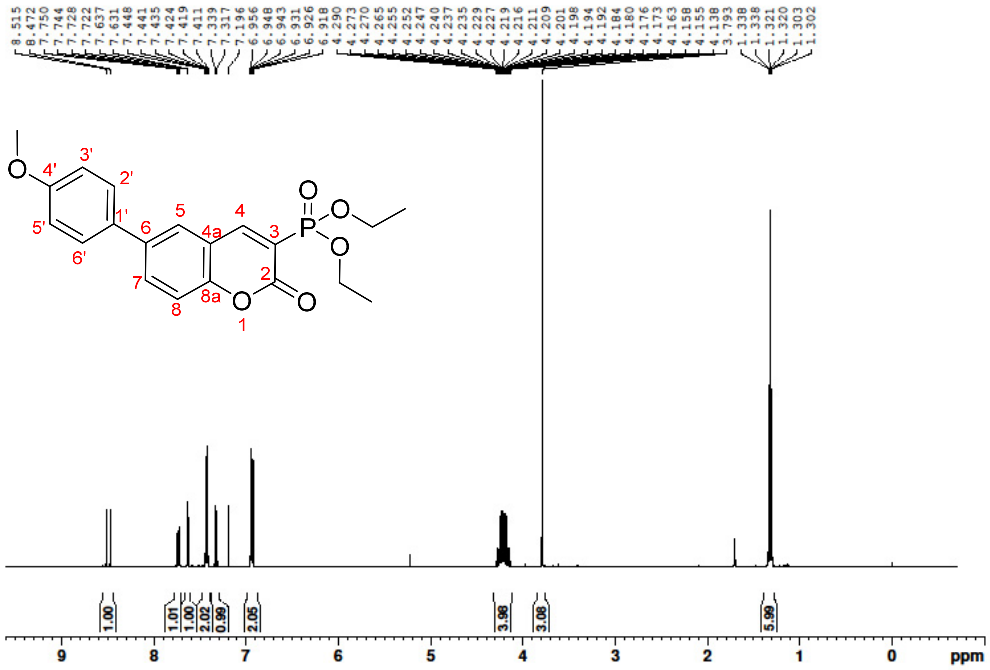


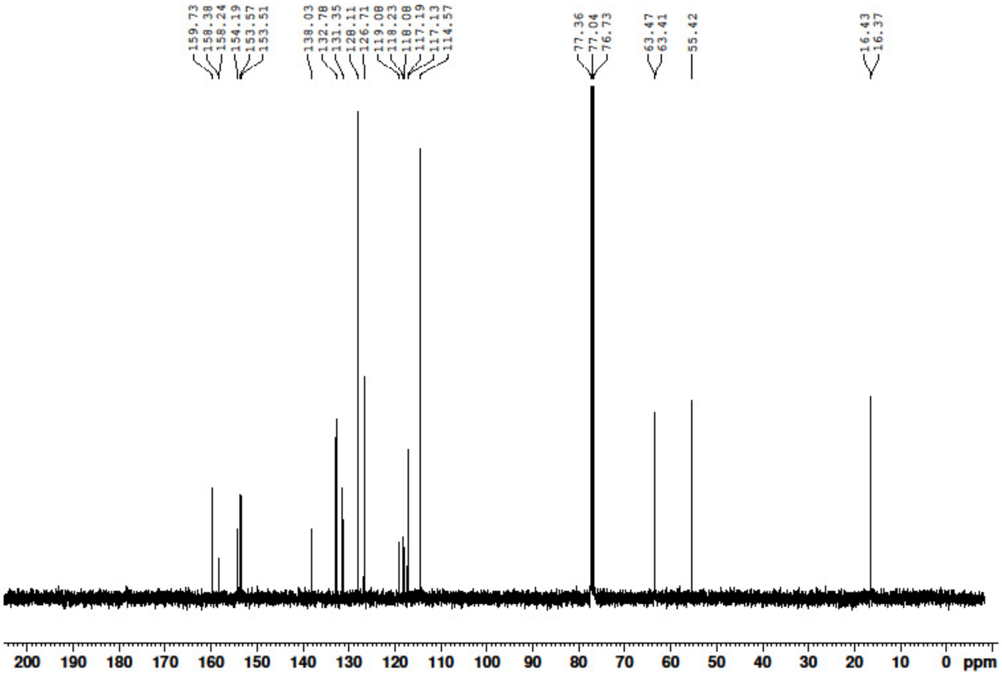

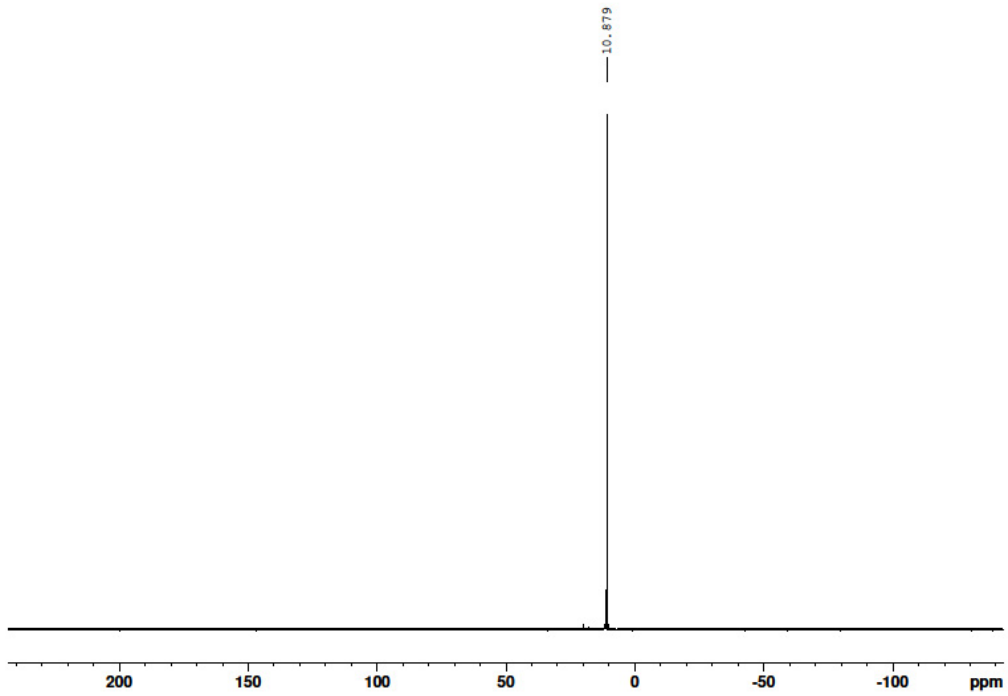
10.879
200
150
100
50
0
-50
-100
ppm

$^{1}$H, $^{13}$C, $^{19}$F and $^{31}$P NMR spectra of compound **3d**

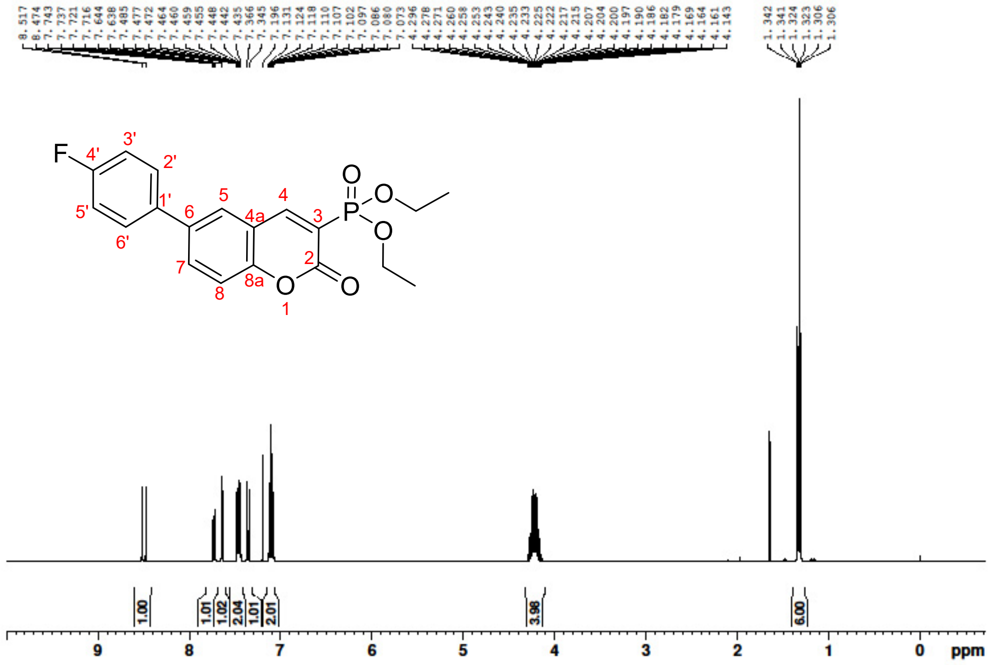


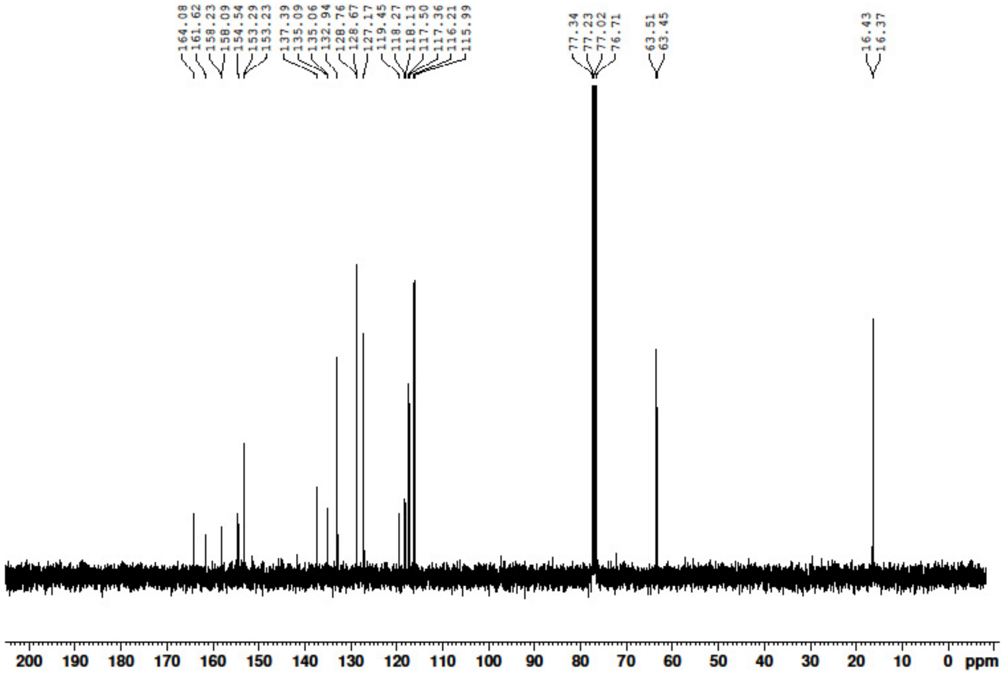

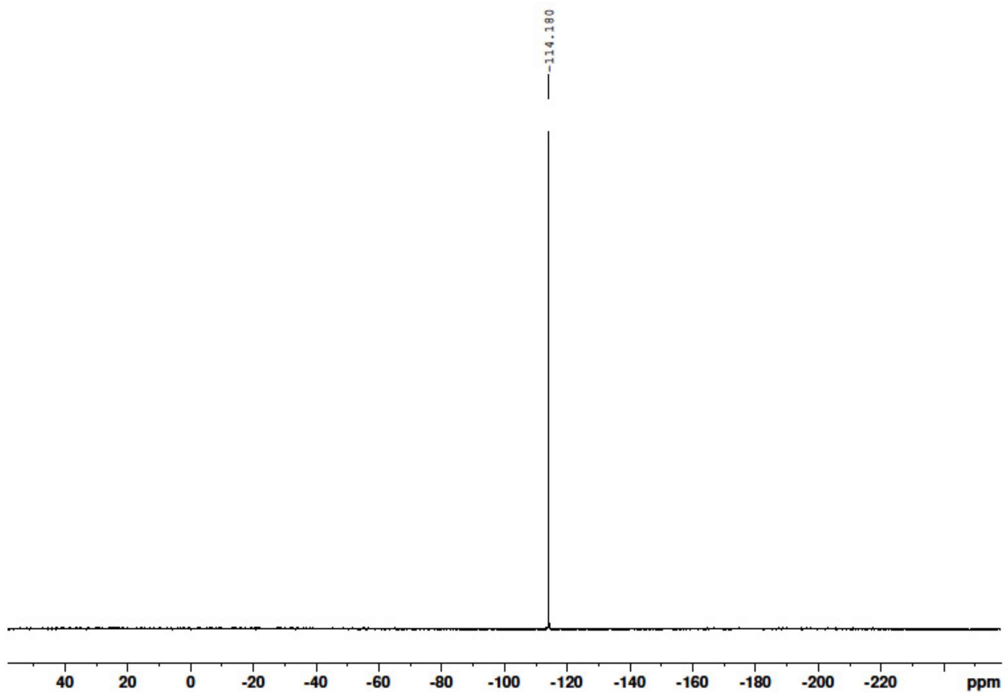
-114.180
40
20
0
-20
-40
-60
-80
-100
-120
-140
-160
-180
-200
-220
ppm

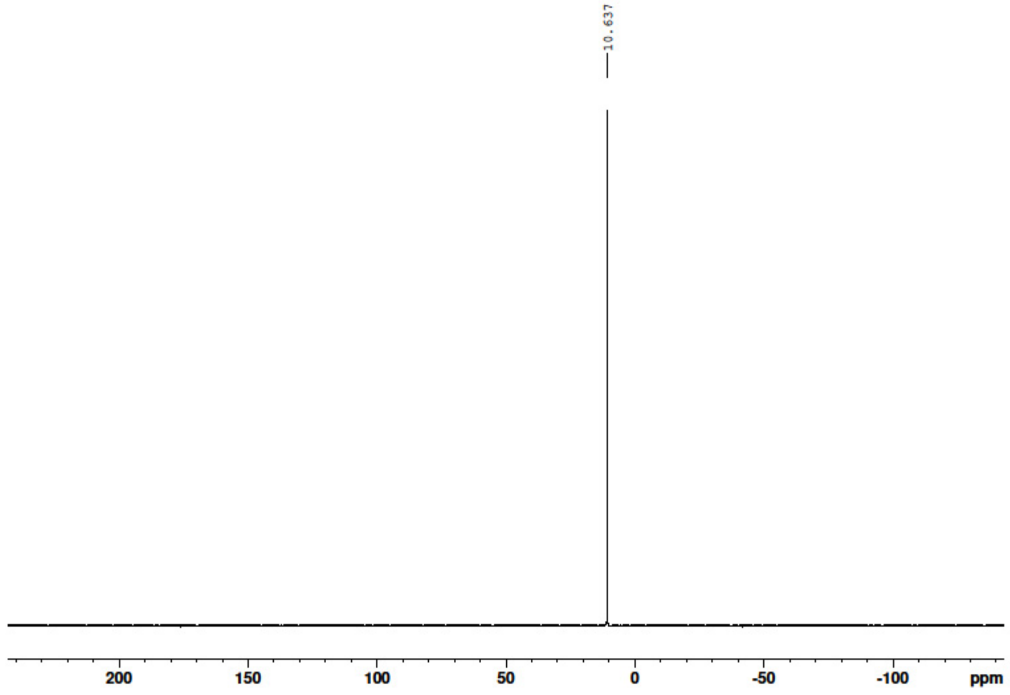
10.637
200
150
100
50
0
-50
-100
ppm

$^{1}H$, $^{13}C$ and $^{31}P$ NMR spectra of compound **3e**

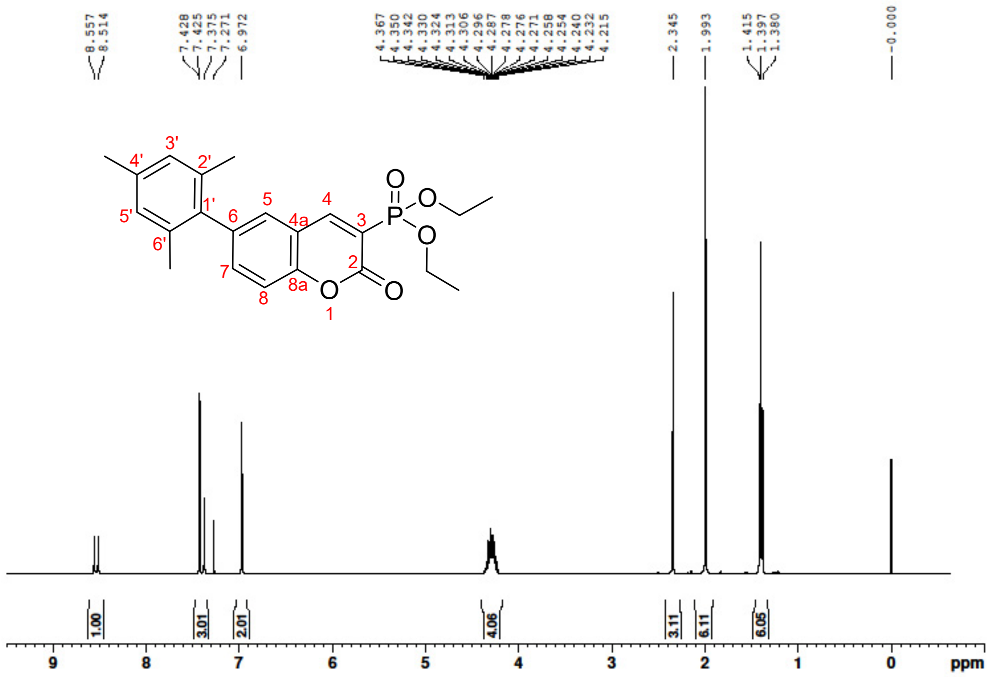


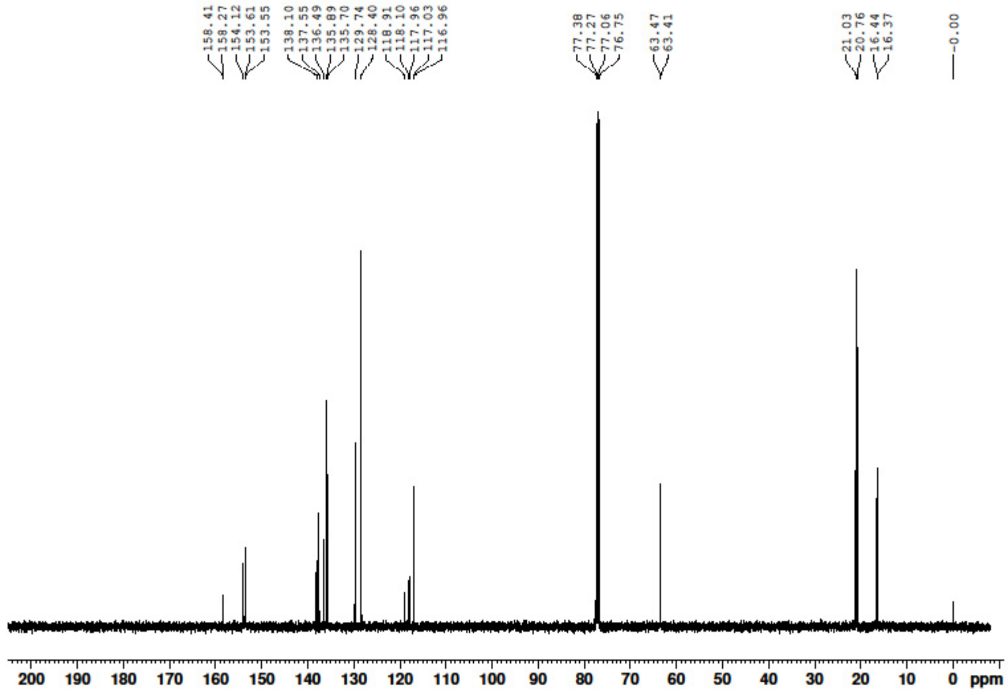

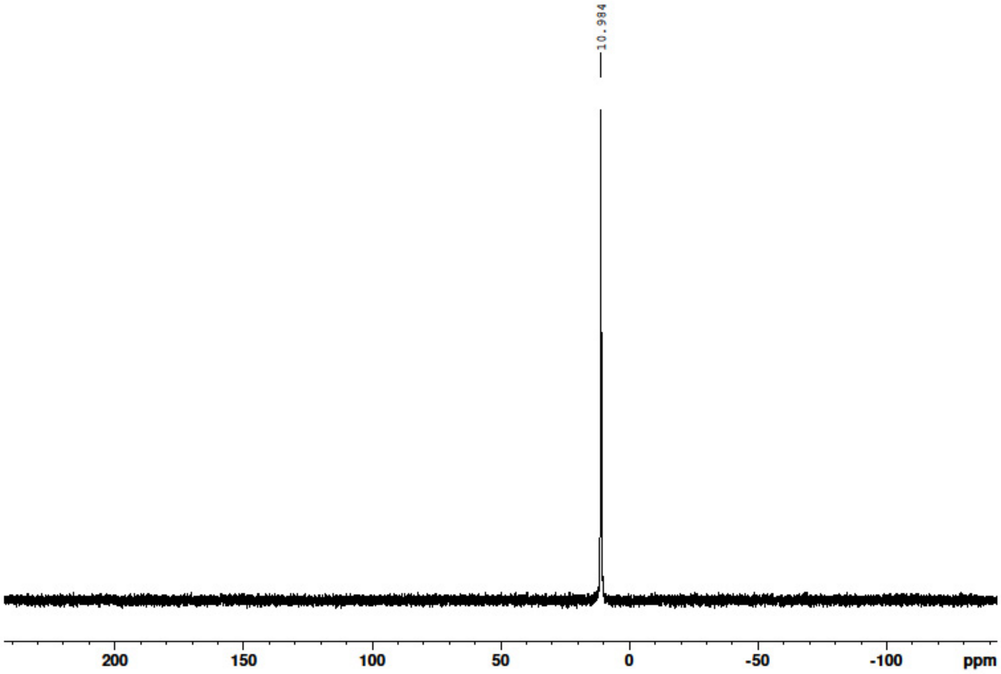
10.984
200
150
100
50
0
-50
-100
ppm

$^{1}H$ and $^{13}C$ NMR spectra of compound **9a**

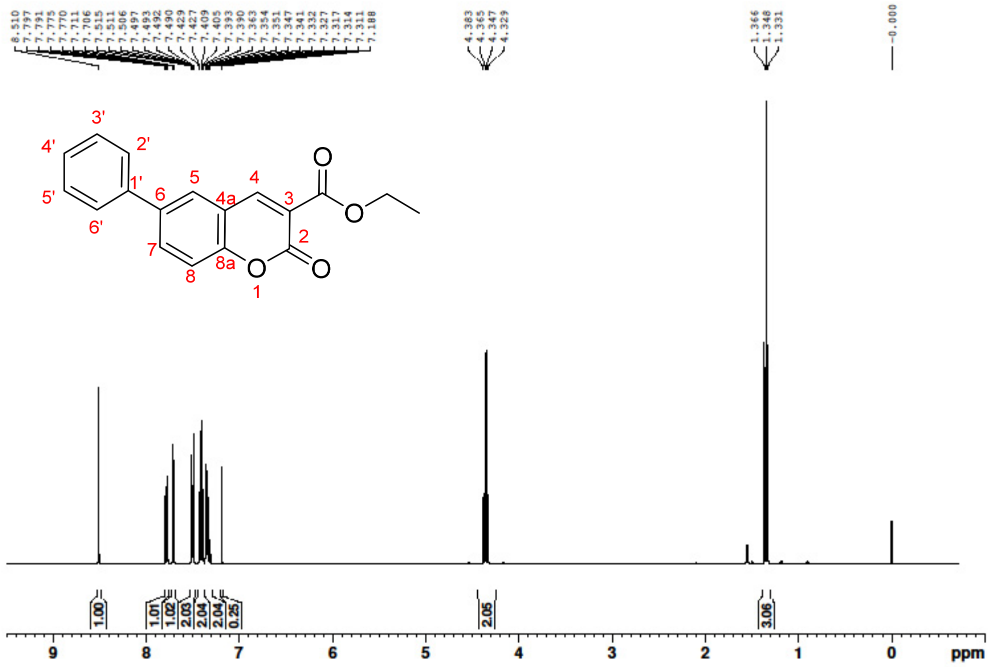


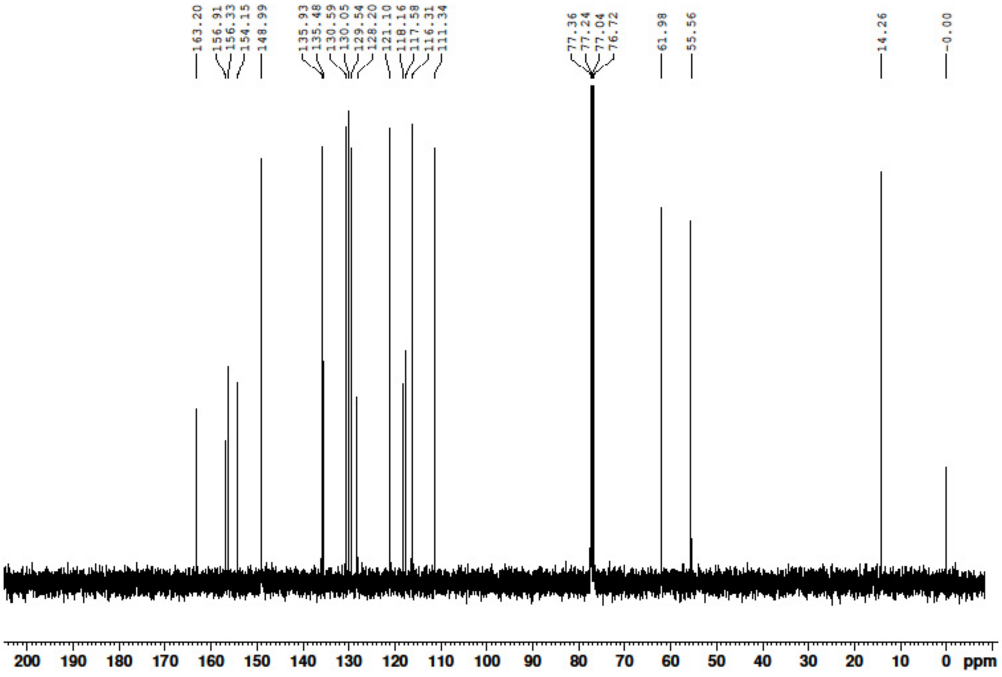

$^1$H and $^{13}$C NMR spectra of compound **9b**

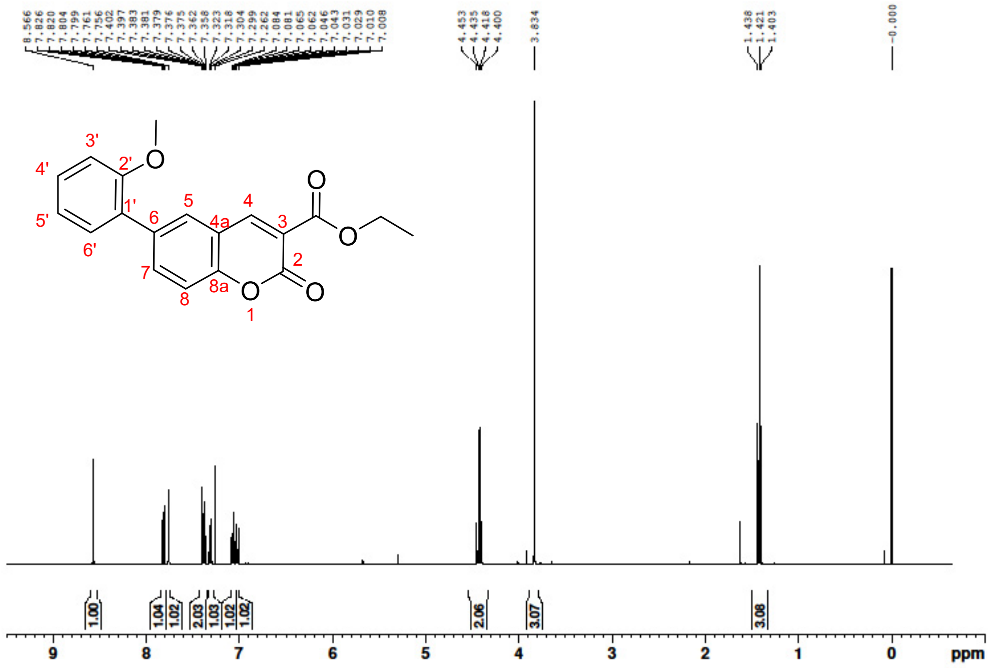

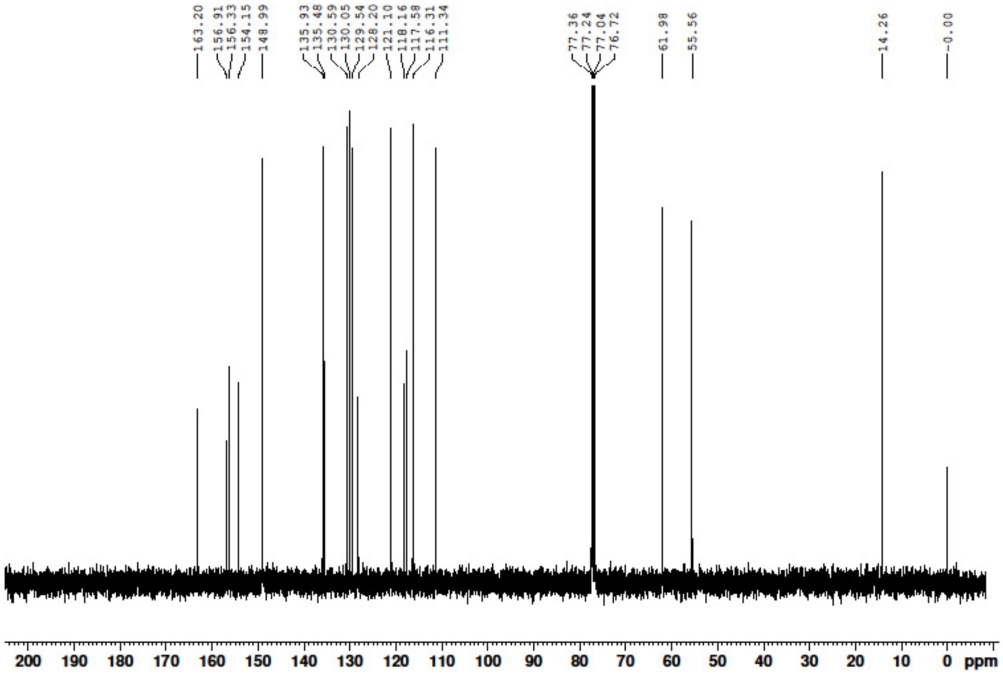

$^{1}H$ and $^{13}C$ NMR spectra of compound **9c**

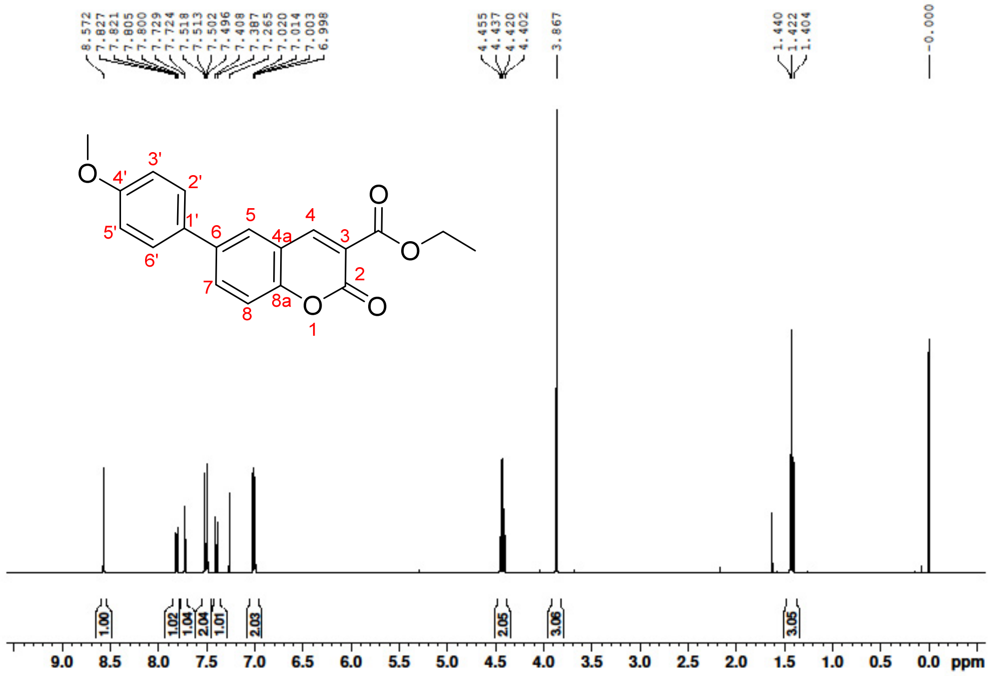


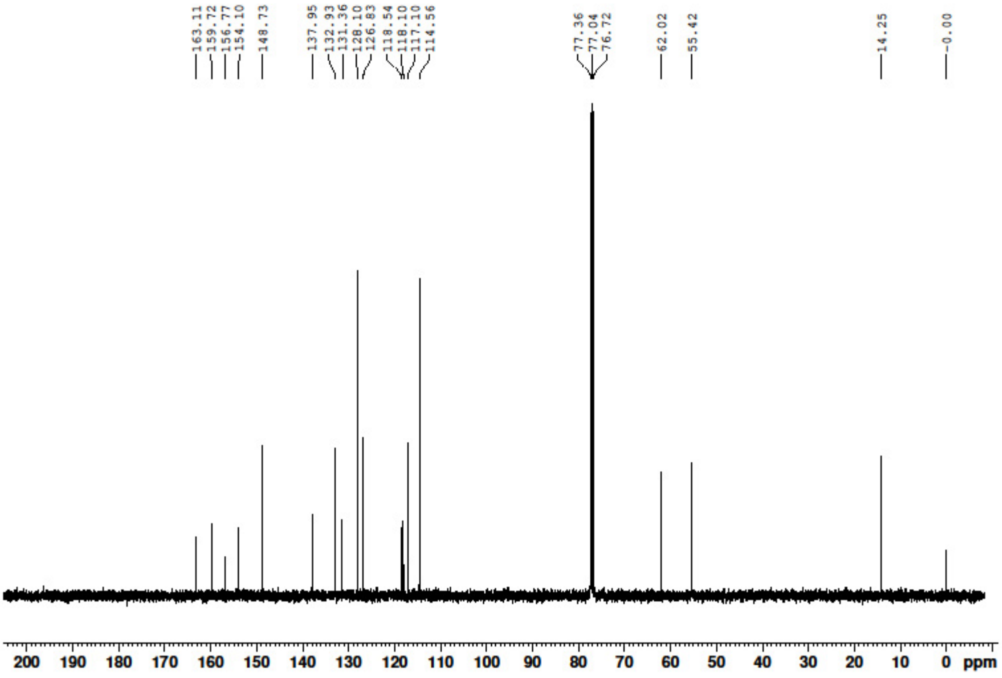

$^{1}$H, $^{13}$C and $^{19}$F NMR spectra of compound **9d**

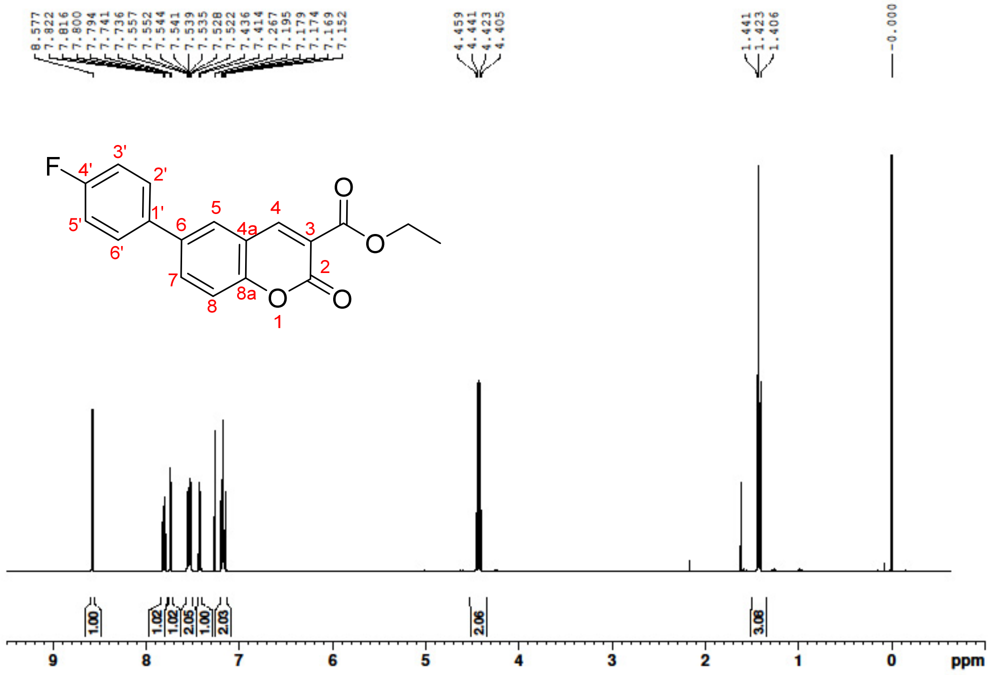


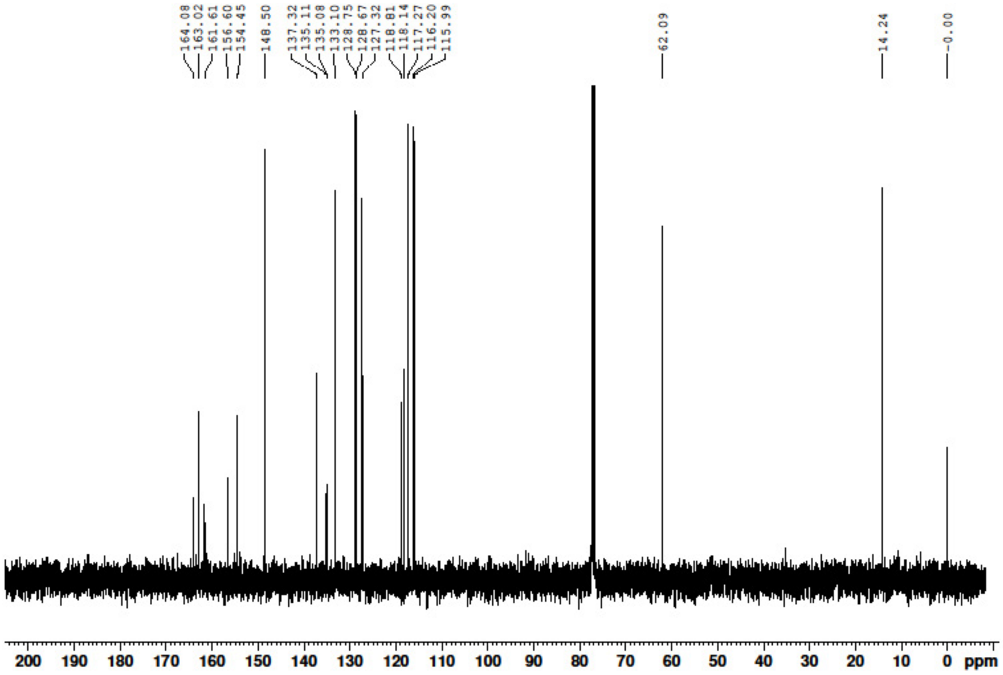

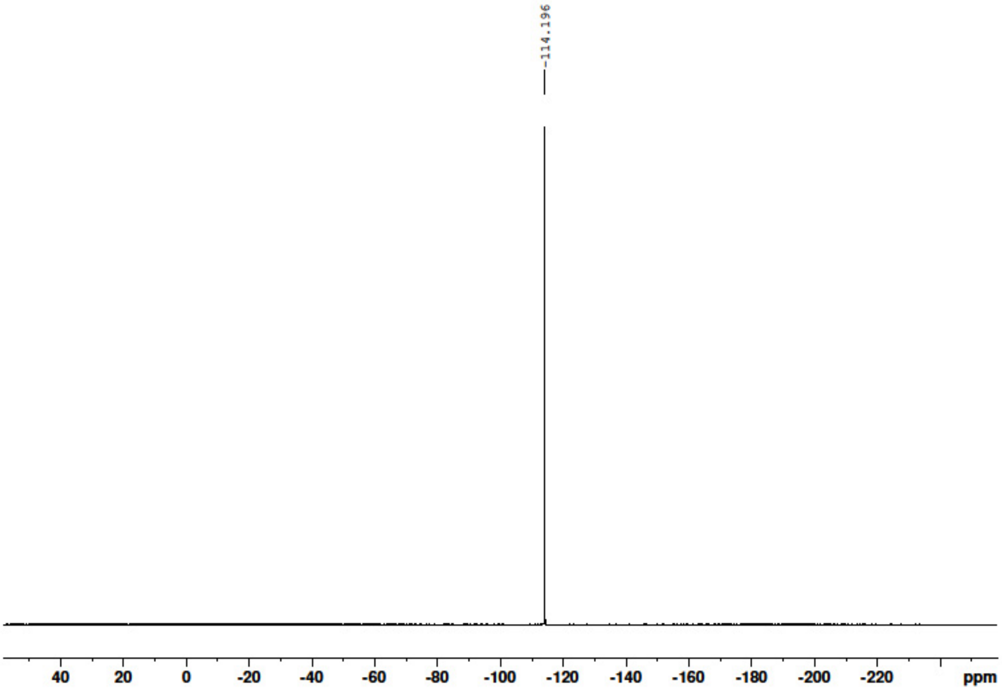
-114.196
40
20
0
-20
-40
-60
-80
-100
-120
-140
-160
-180
-200
-220
ppm

$^{1}$H and $^{13}$C NMR spectra of compound **9e**

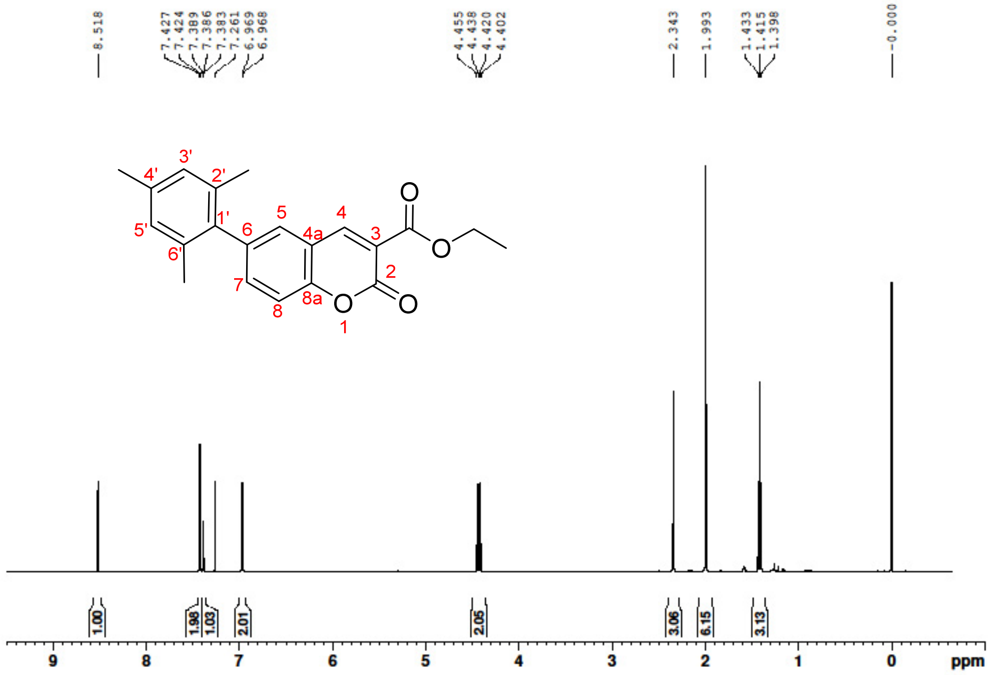


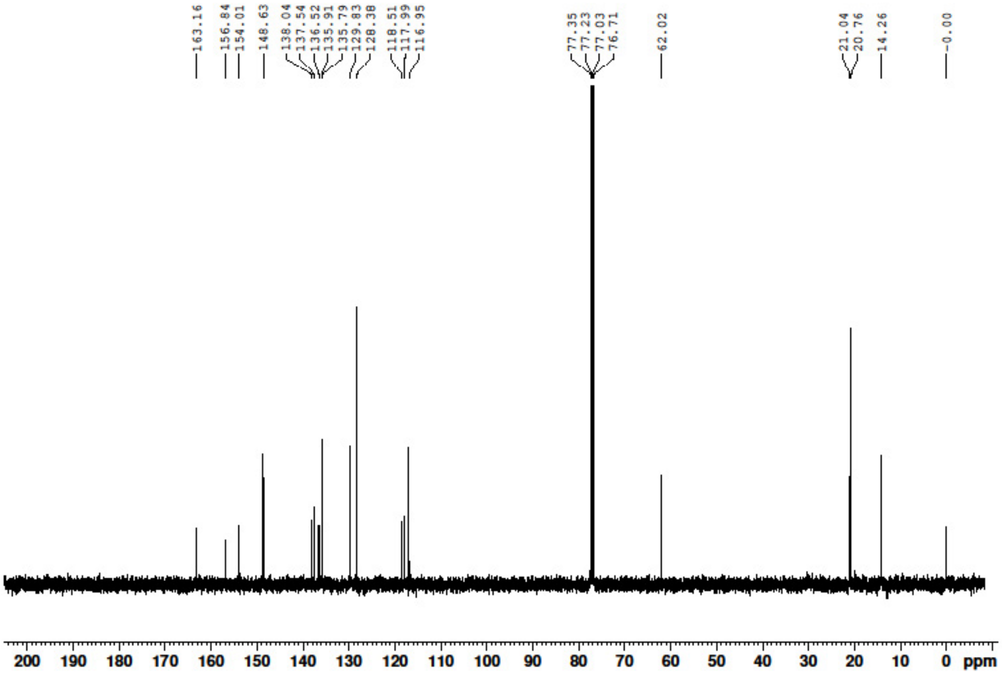

$^{1}$H and $^{13}$C NMR spectra of compound **10a**

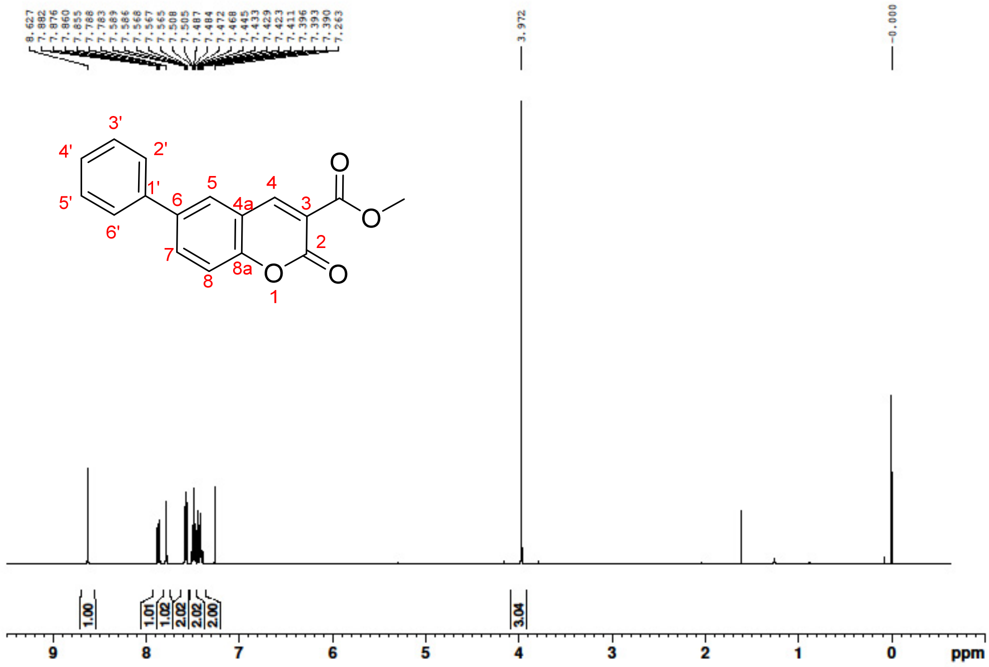


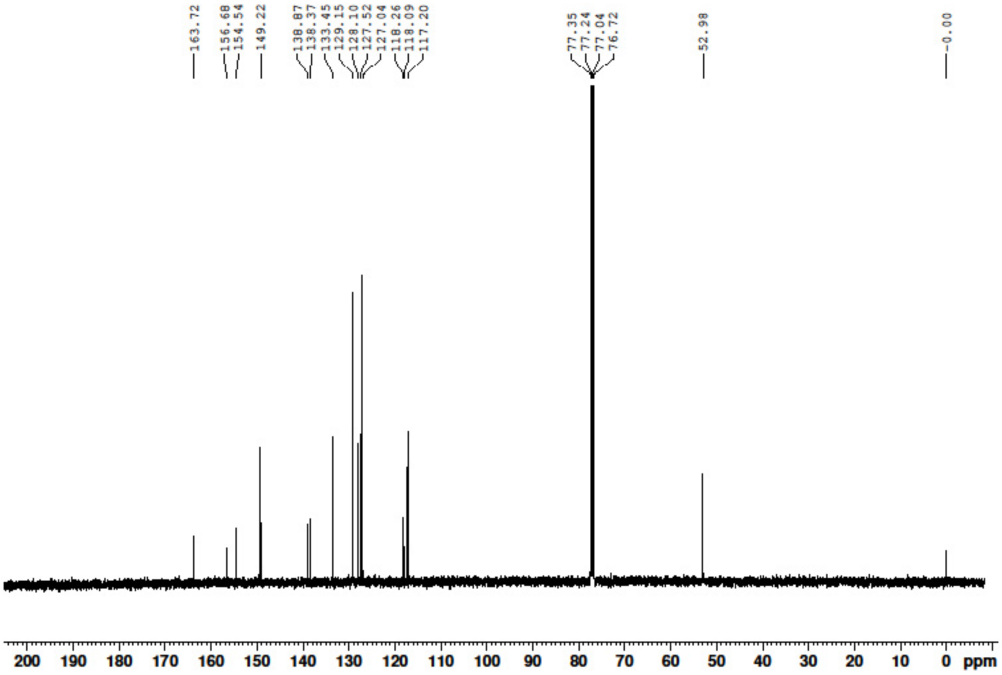

$^{1}H$ and $^{13}C$ NMR spectra of compound **10b**

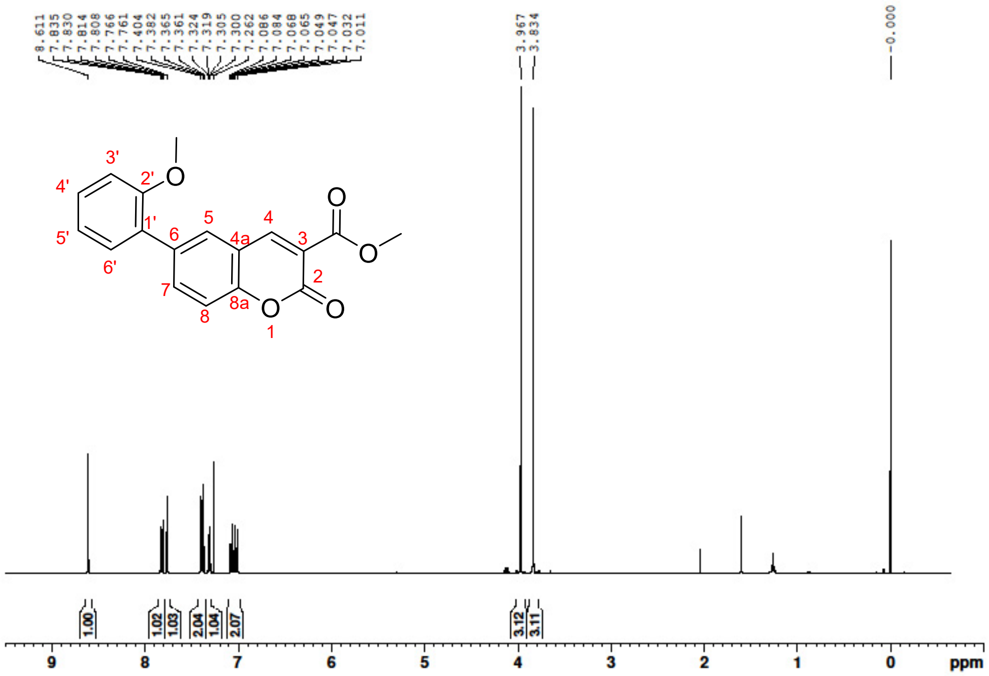


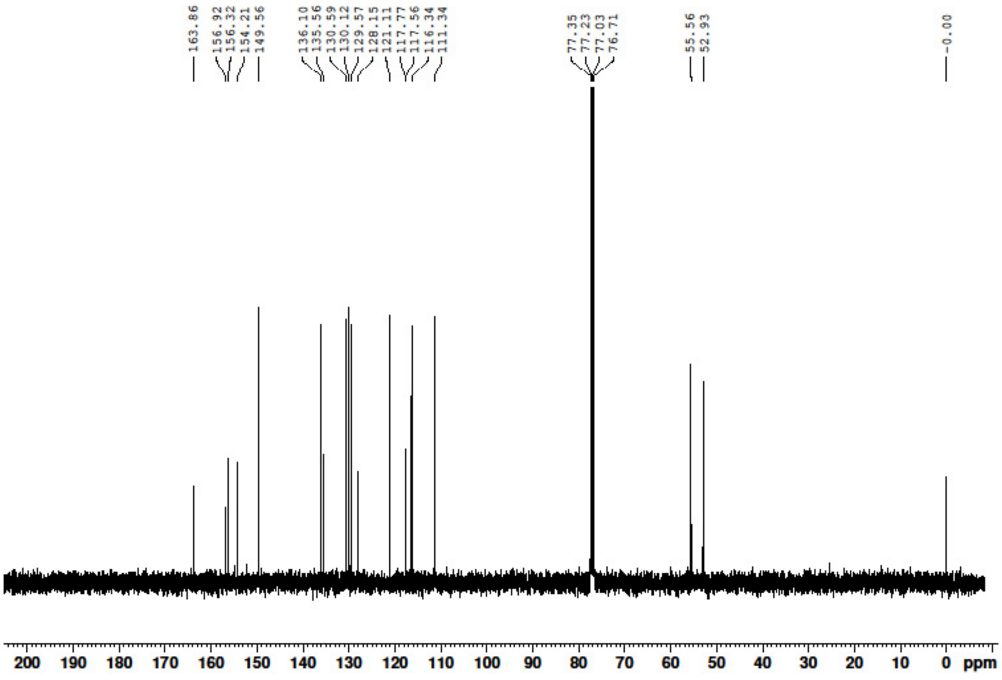

$^{1}$H and $^{13}$C NMR spectra of compound **10c**

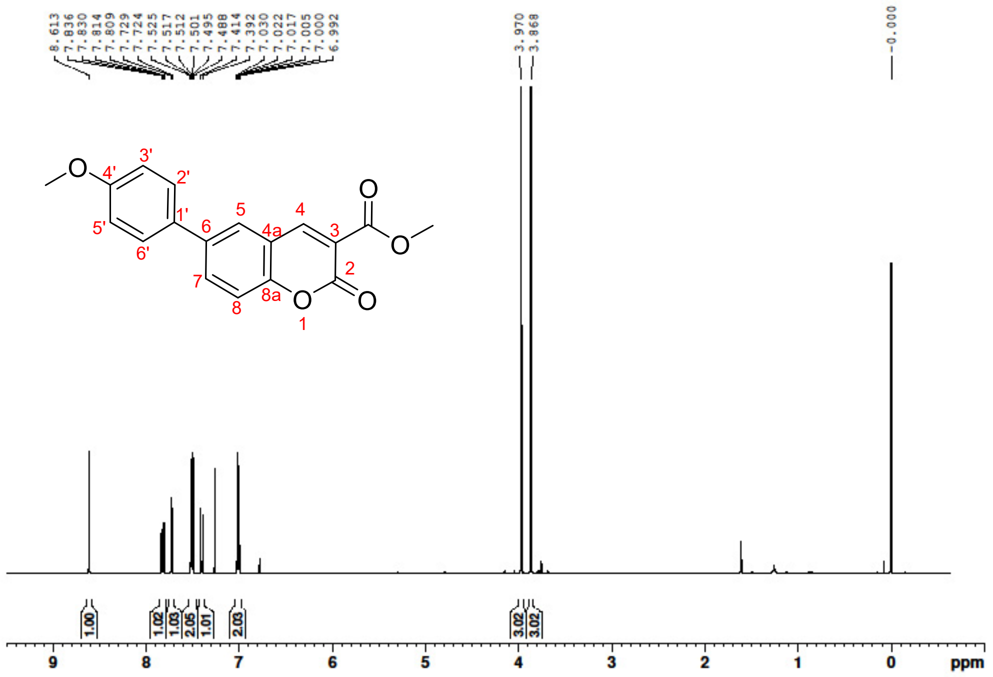

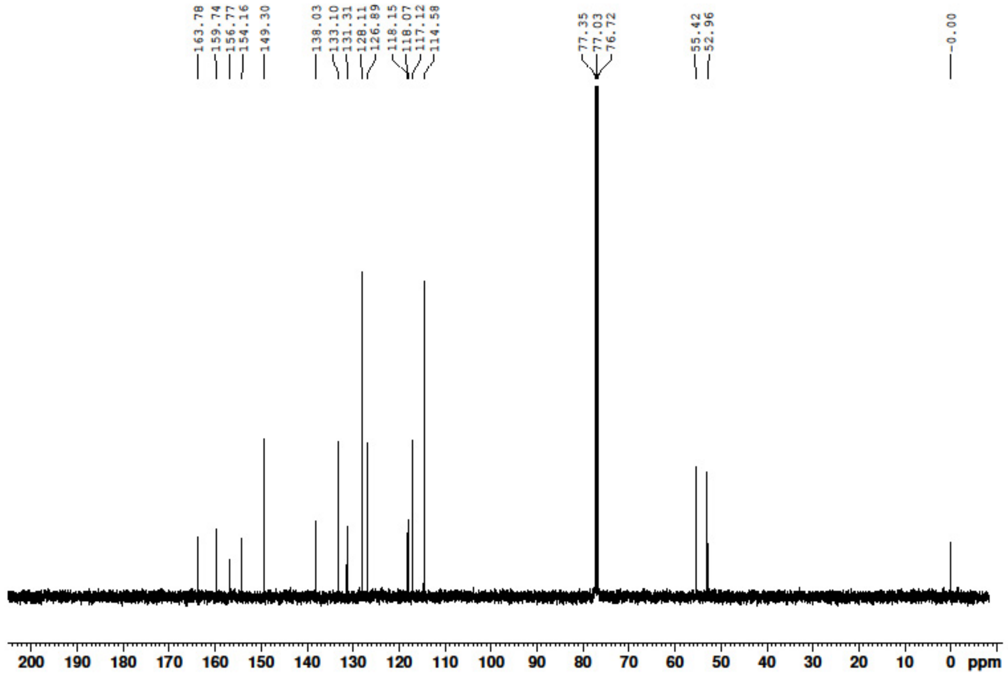

$^{1}H$, $^{13}C$ and $^{19}F$ NMR spectra of compound **10d**

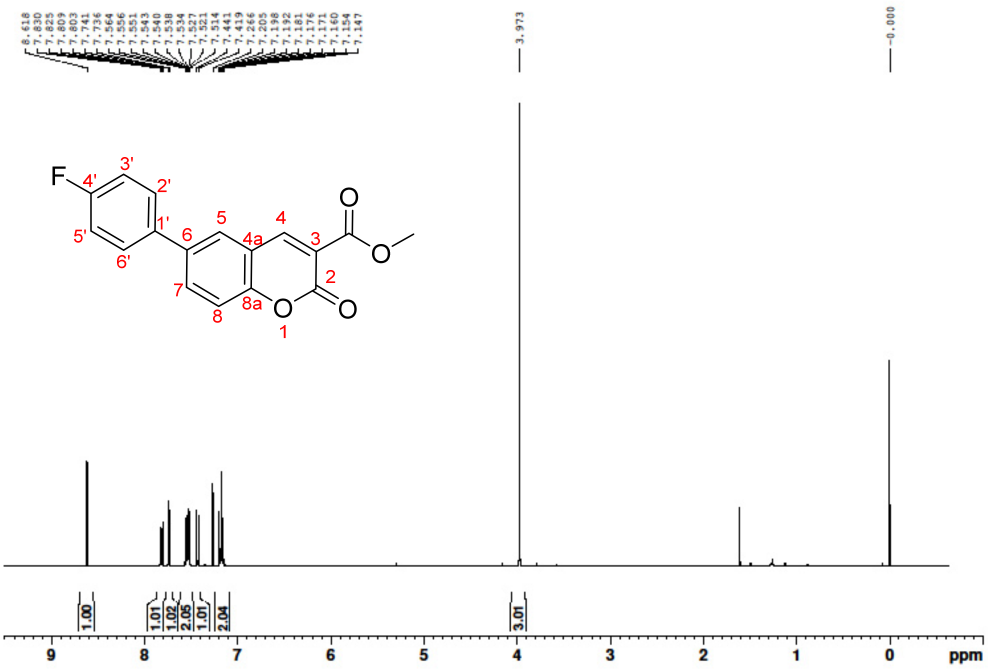


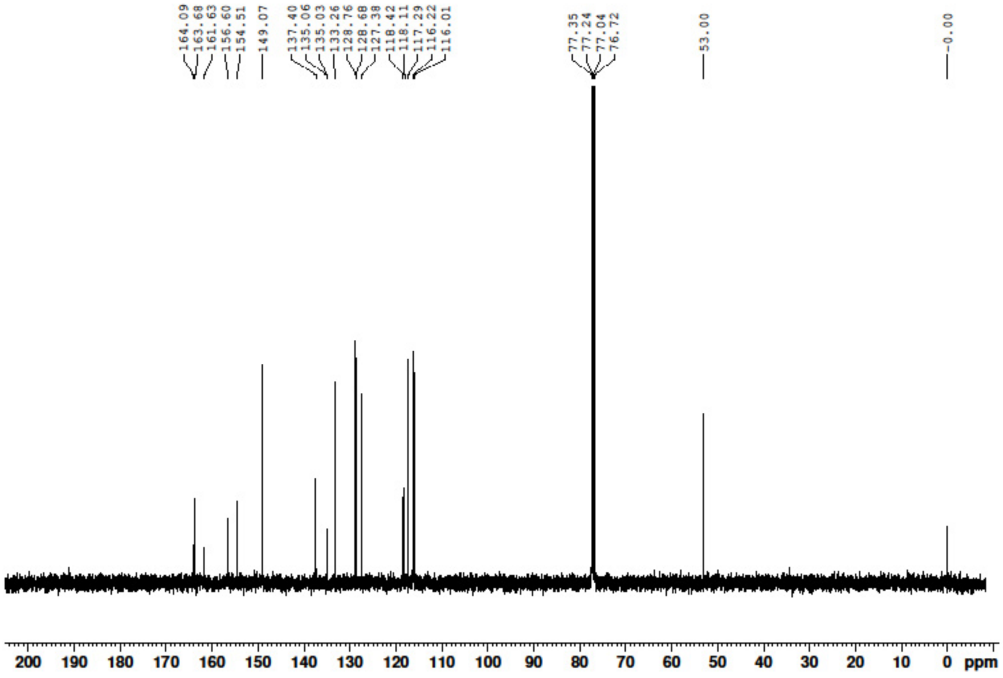

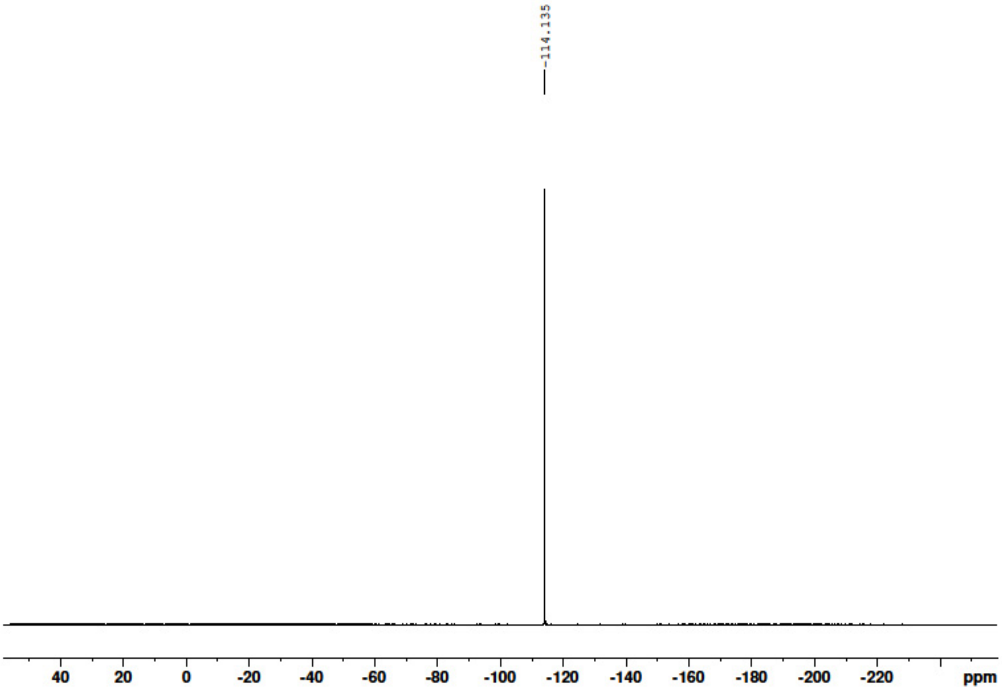
-114.135
40
20
0
-20
-40
-60
-80
-100
-120
-140
-160
-180
-200
-220
ppm

$^{1}$H and $^{13}$C NMR spectra of compound **10e**

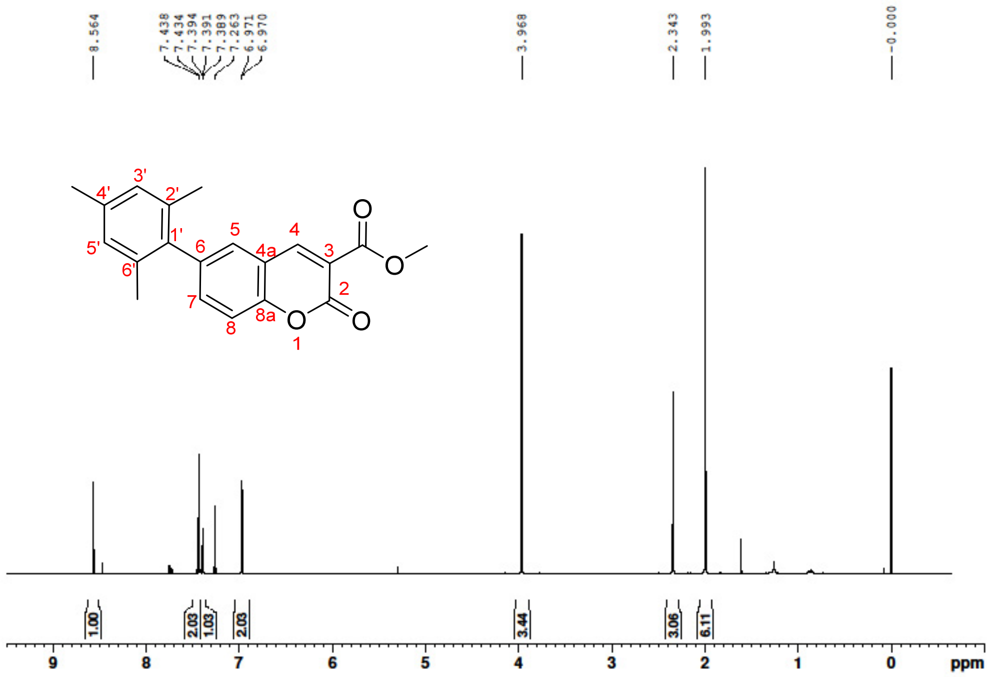

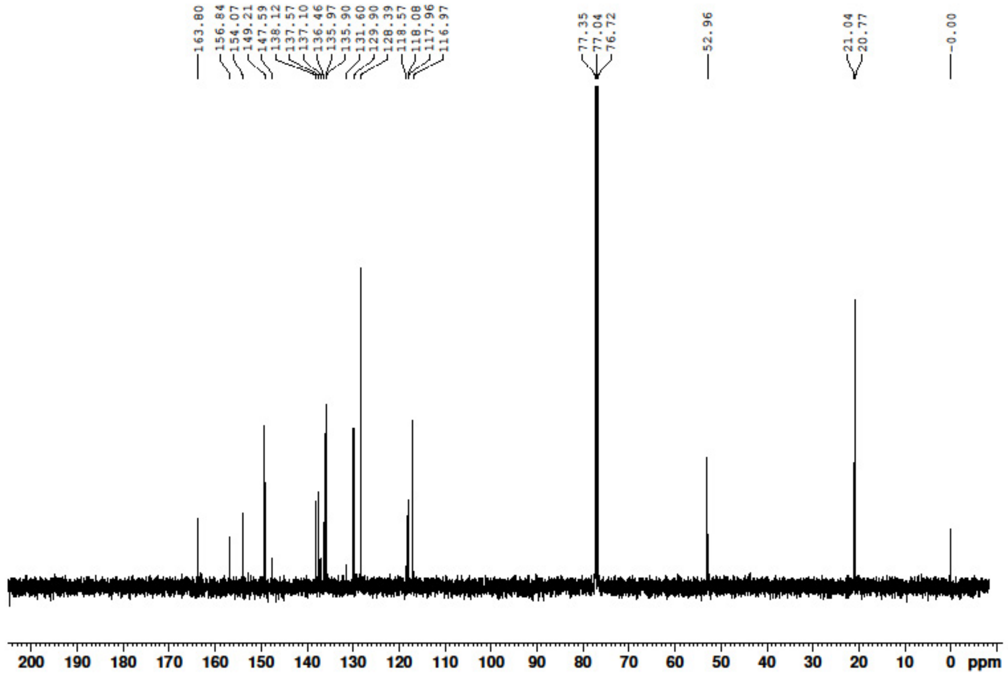

$^{1}H$, $^{13}C$ and $^{31}P$ NMR spectra of compound **11a**

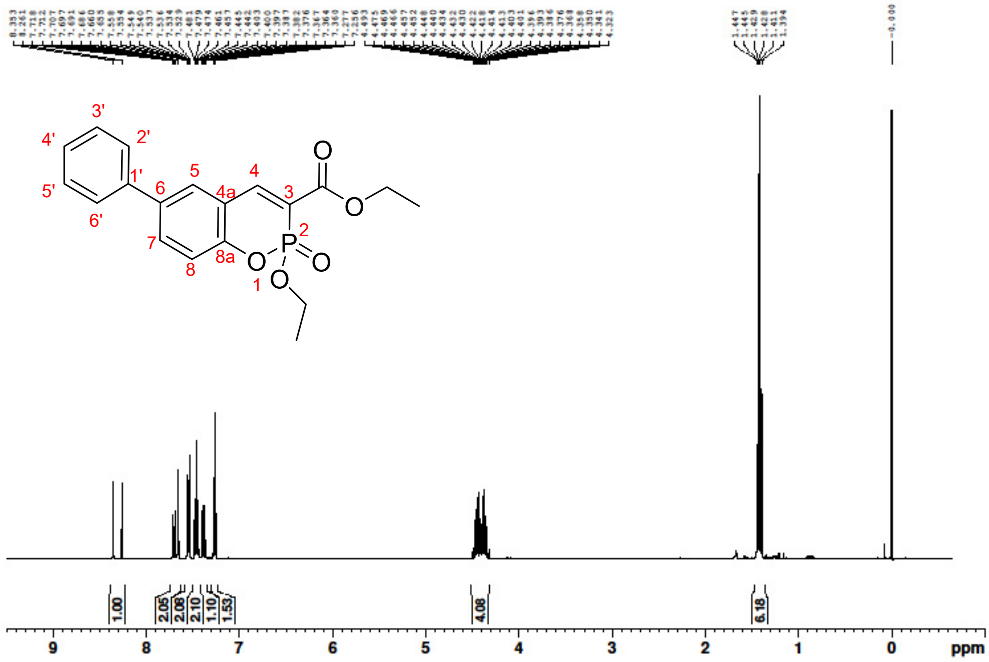


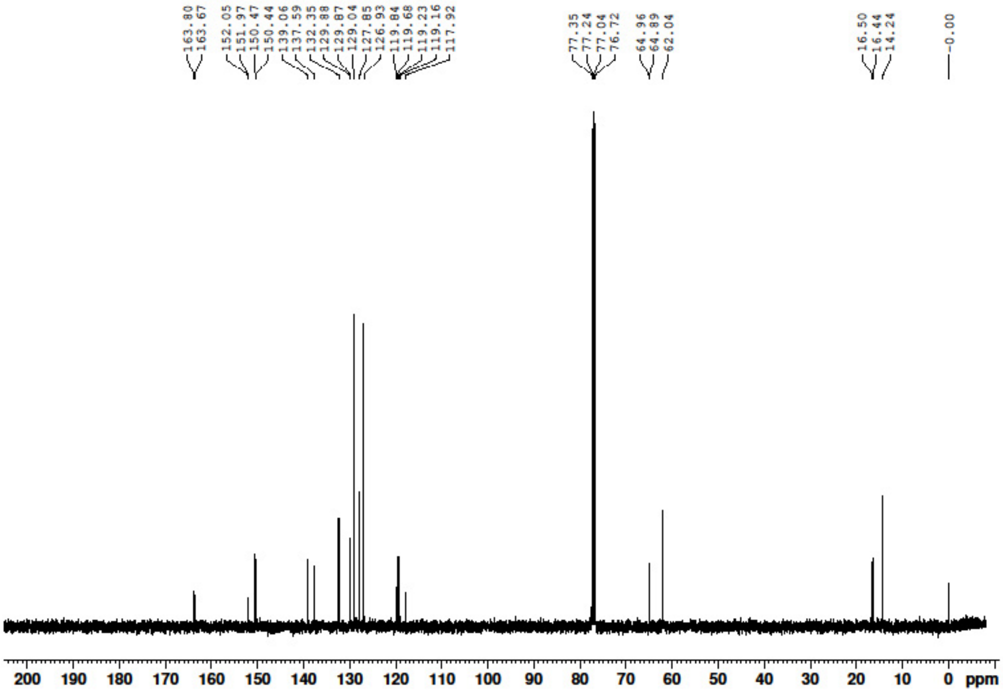

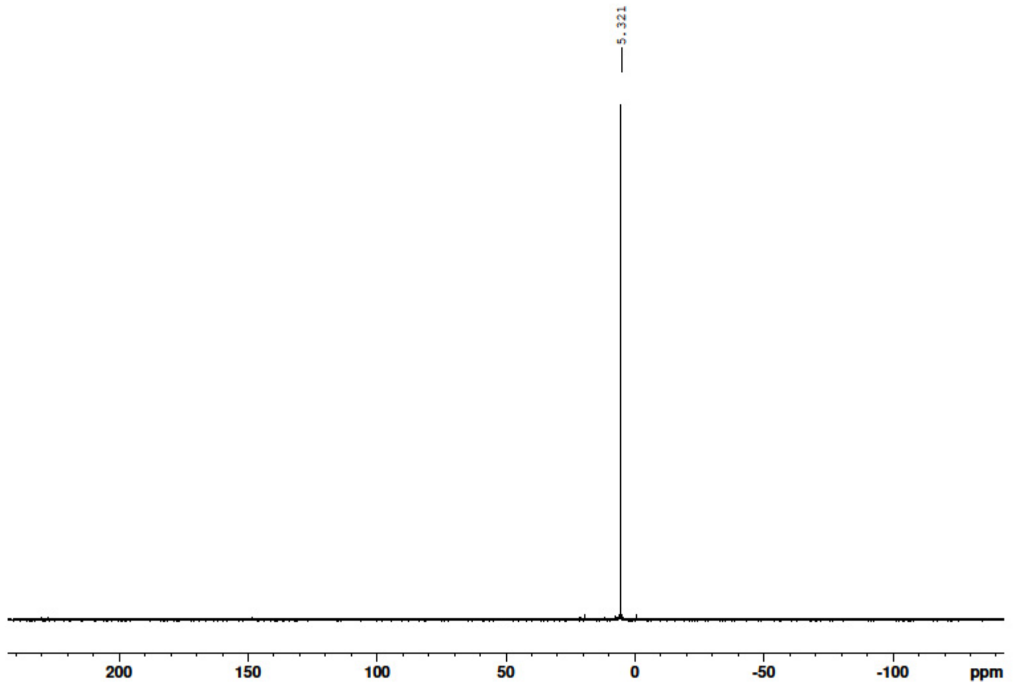

5.321
200
150
100
50
0
-50
-100
ppm

$^{1}$H, $^{13}$C and $^{31}$P NMR spectra of compound **11b**

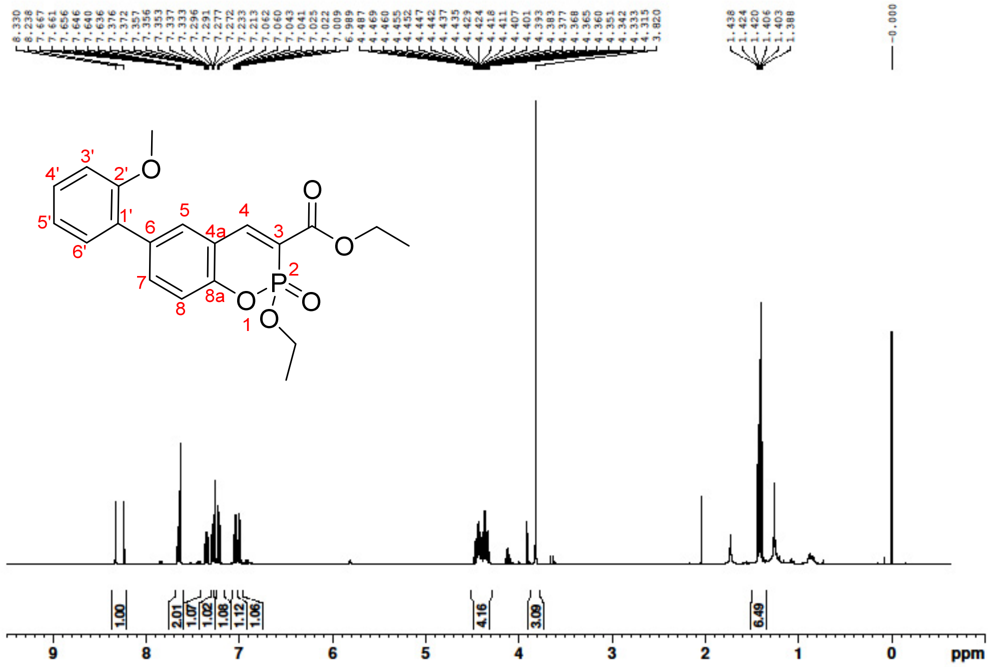


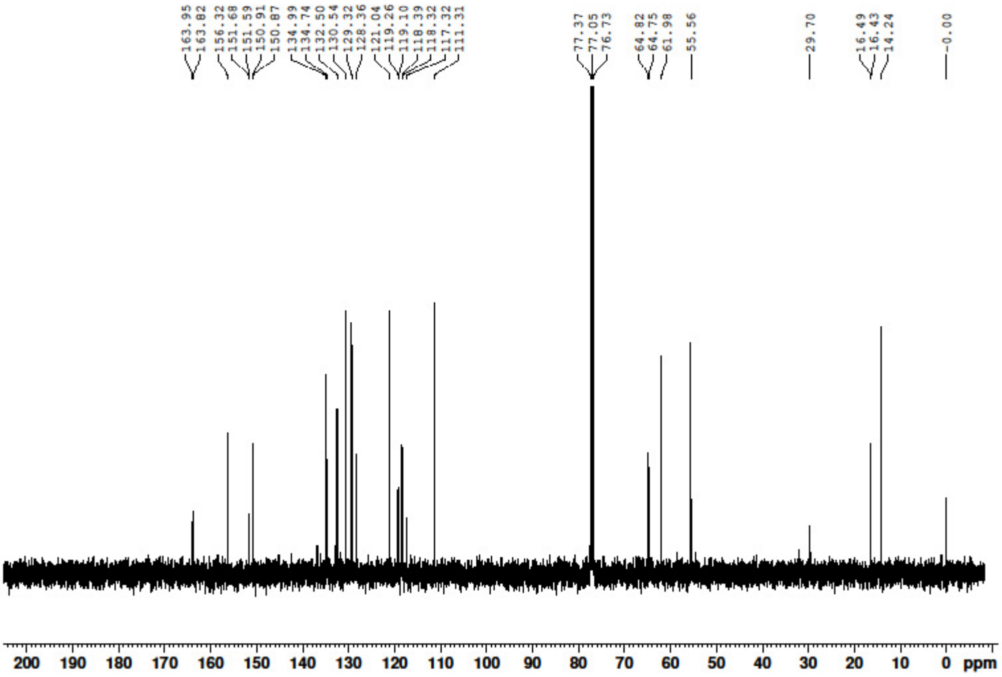

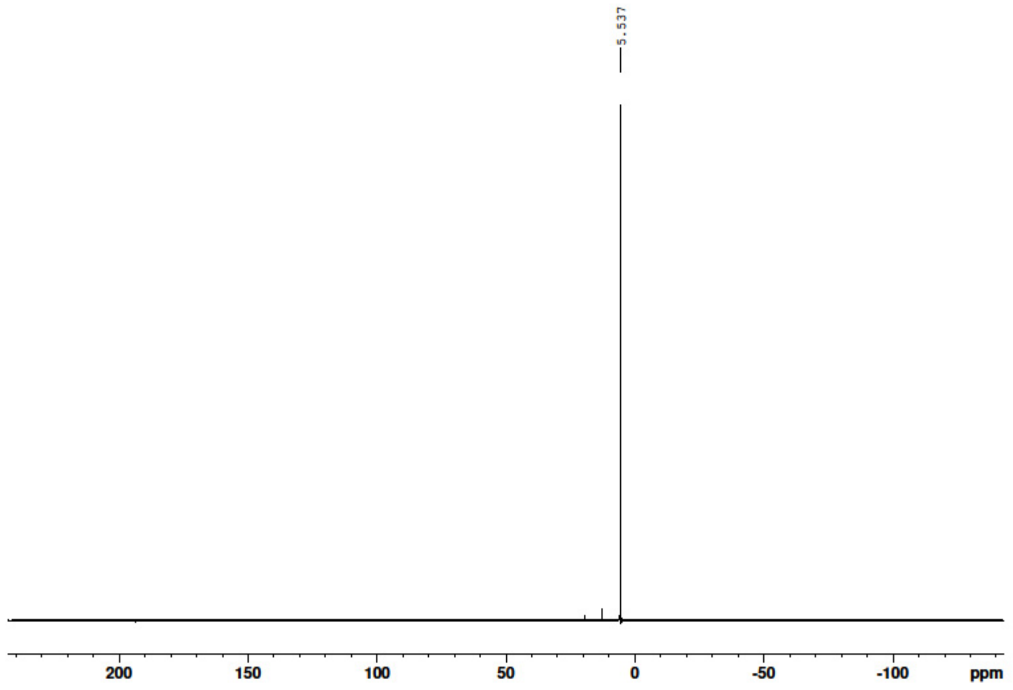

5.537
200
150
100
50
0
-50
-100
ppm

$^{1}H$, $^{13}C$ and $^{31}P$ NMR spectra of compound **11c**

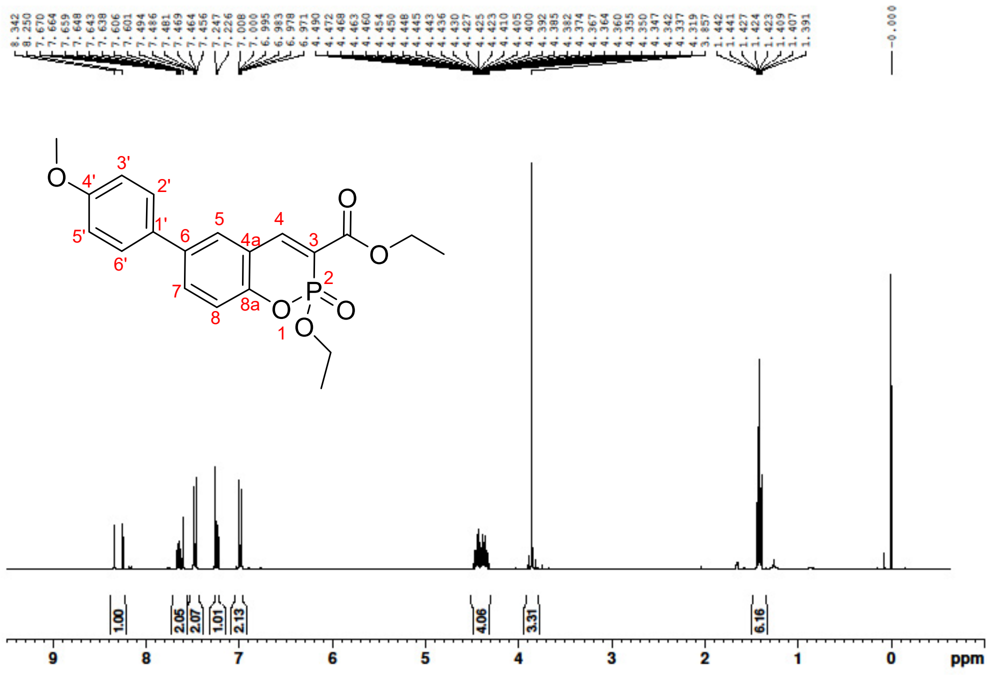


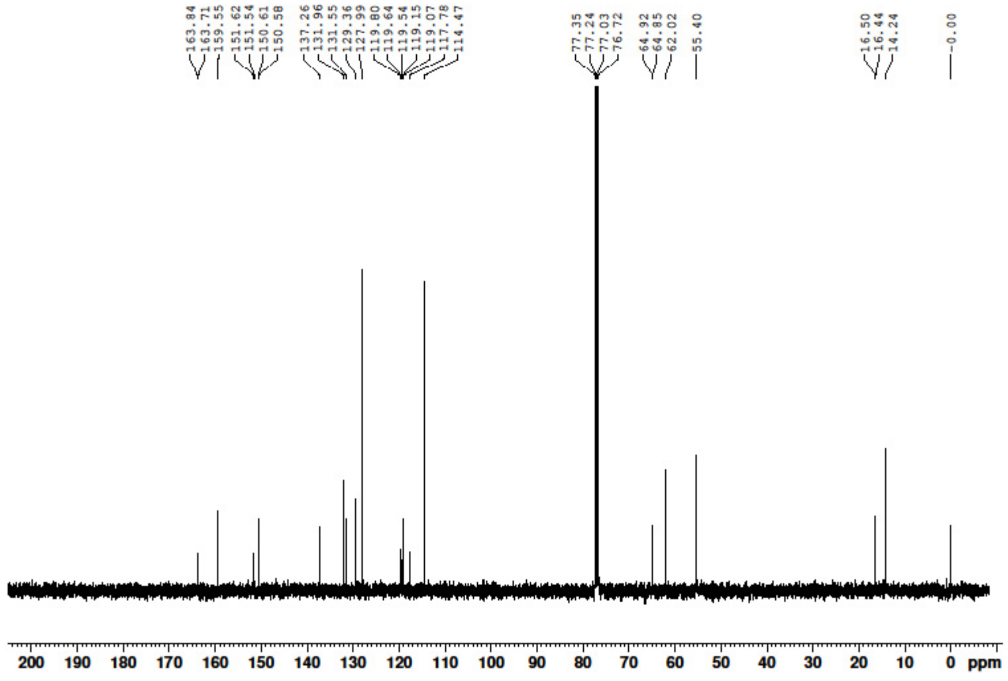

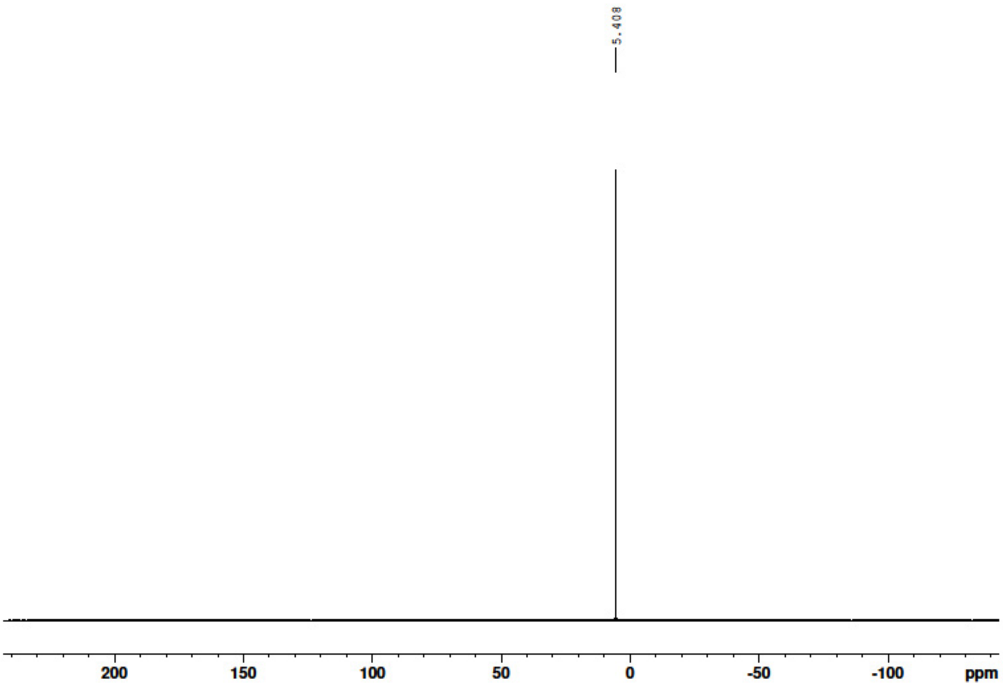

5.408
200
150
100
50
0
-50
-100
ppm

$^{1}$H, $^{13}$C, $^{19}$F and $^{31}$P NMR spectra of compound **11d**

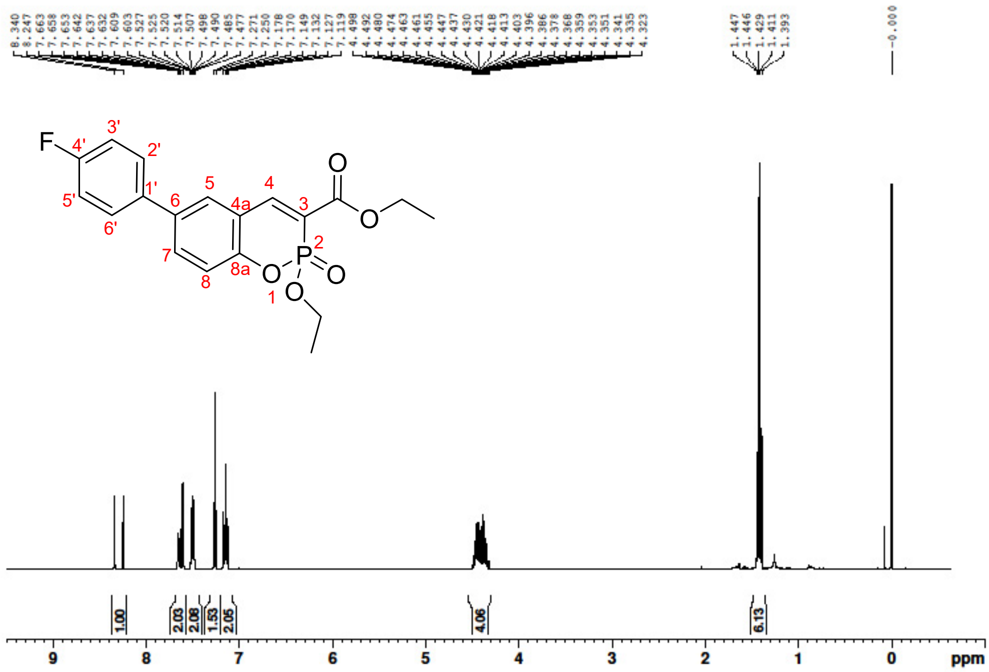


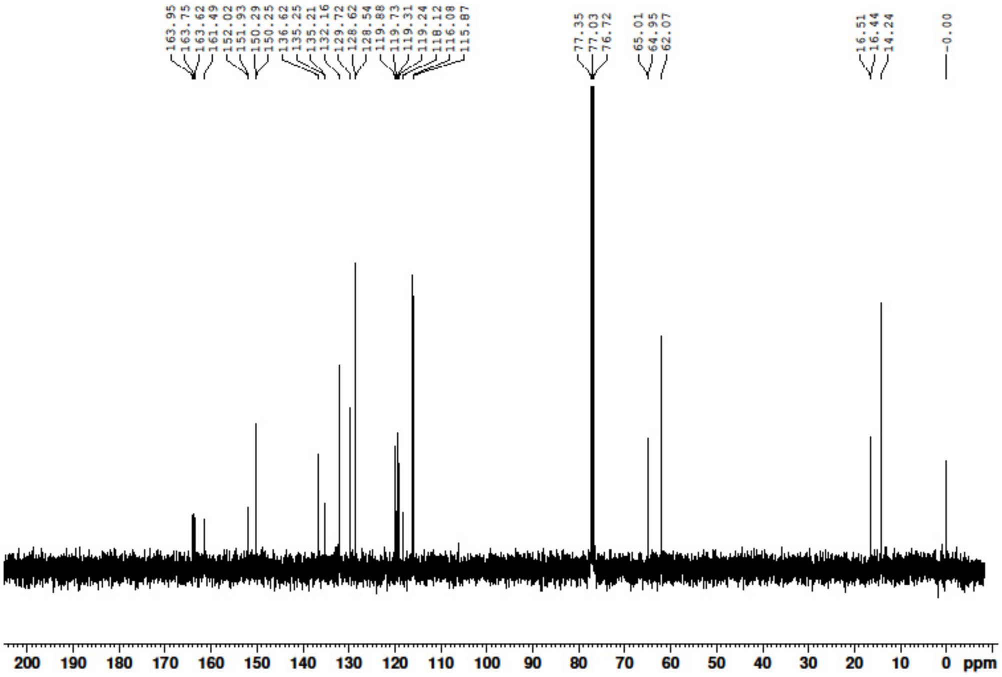

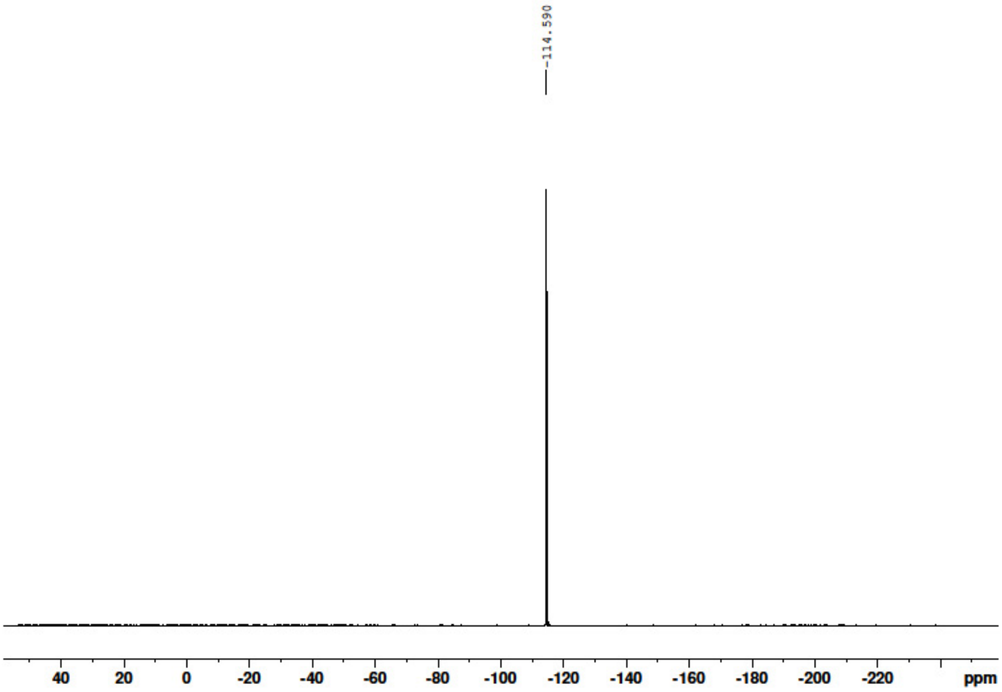
-114.590
40
20
0
-20
-40
-60
-80
-100
-120
-140
-160
-180
-200
-220
ppm

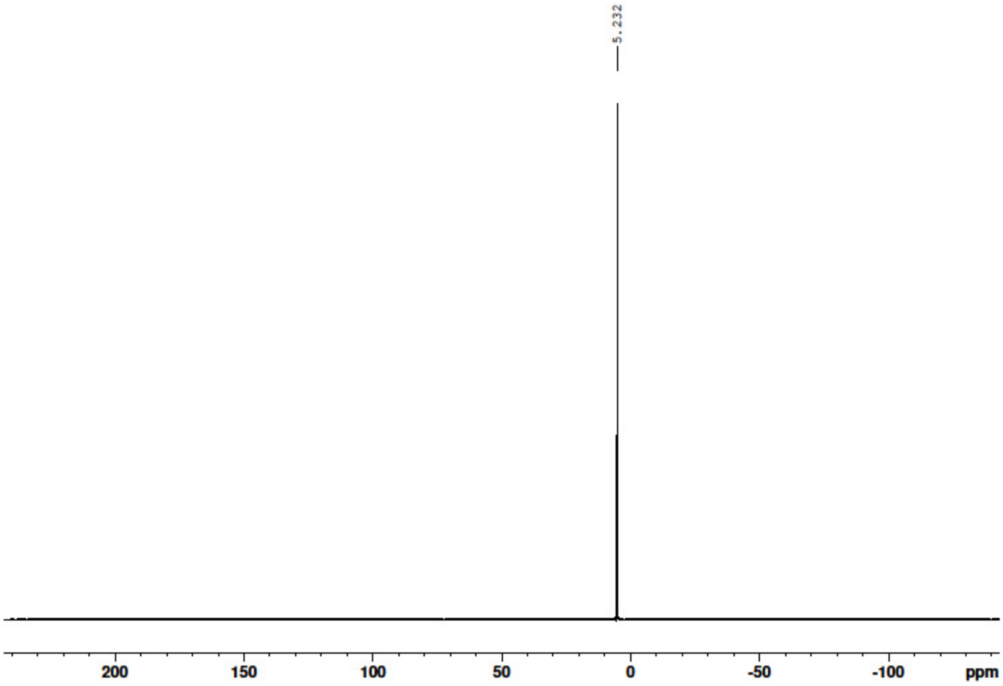
5.232
200
150
100
50
0
-50
-100
ppm

$^{1}$H, $^{13}$C and $^{31}$P NMR spectra of compound **12a**

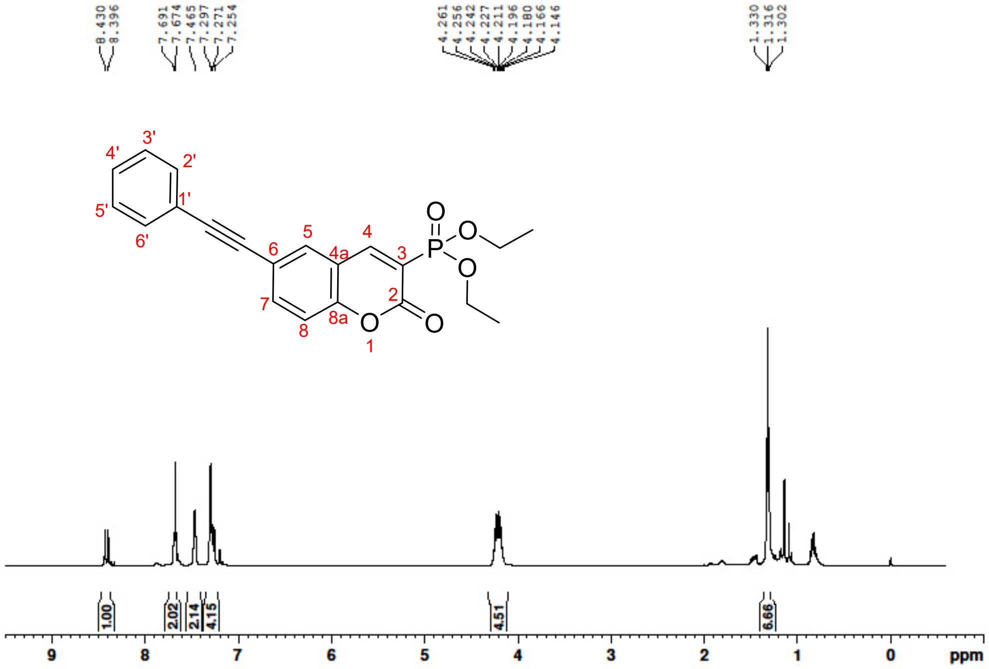


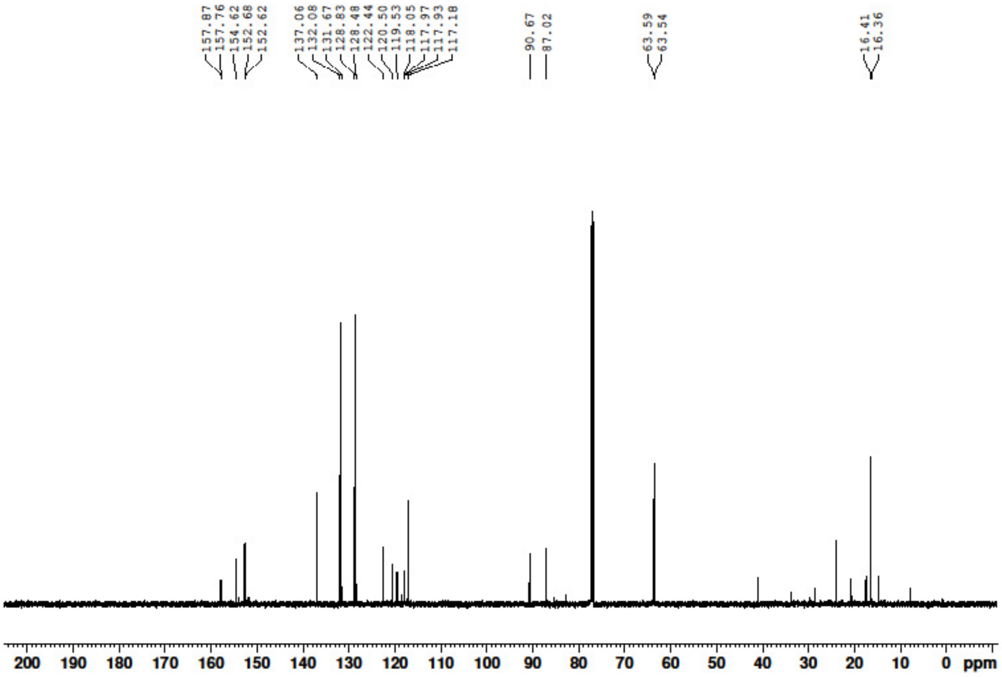

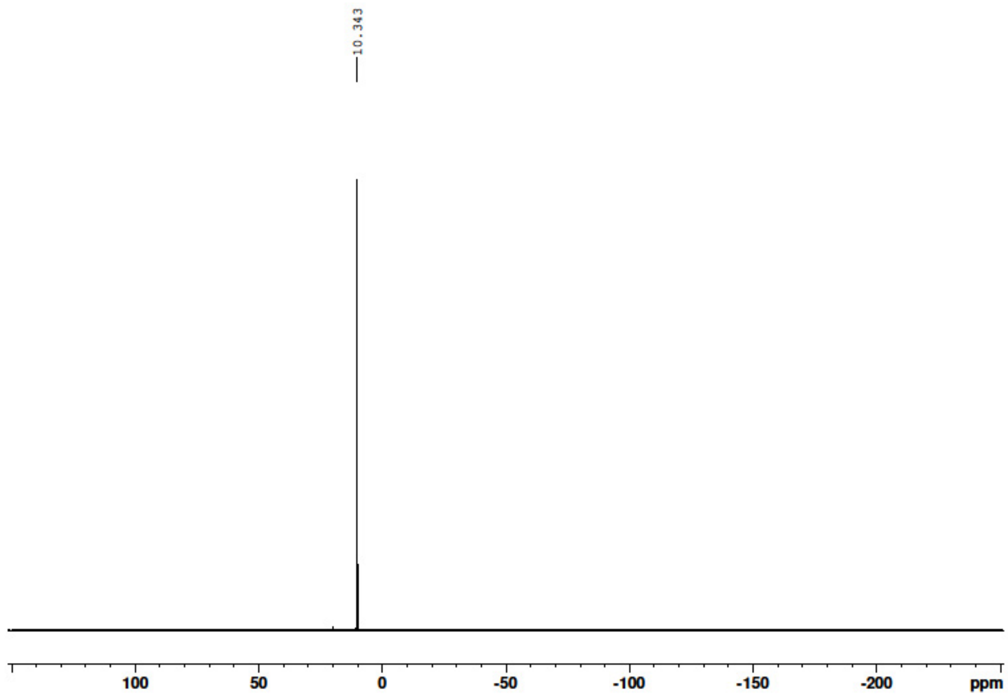
10.343
100
50
0
-50
-100
-150
-200
ppm

$^{1}$H and $^{13}$C NMR spectra of compound **12b**

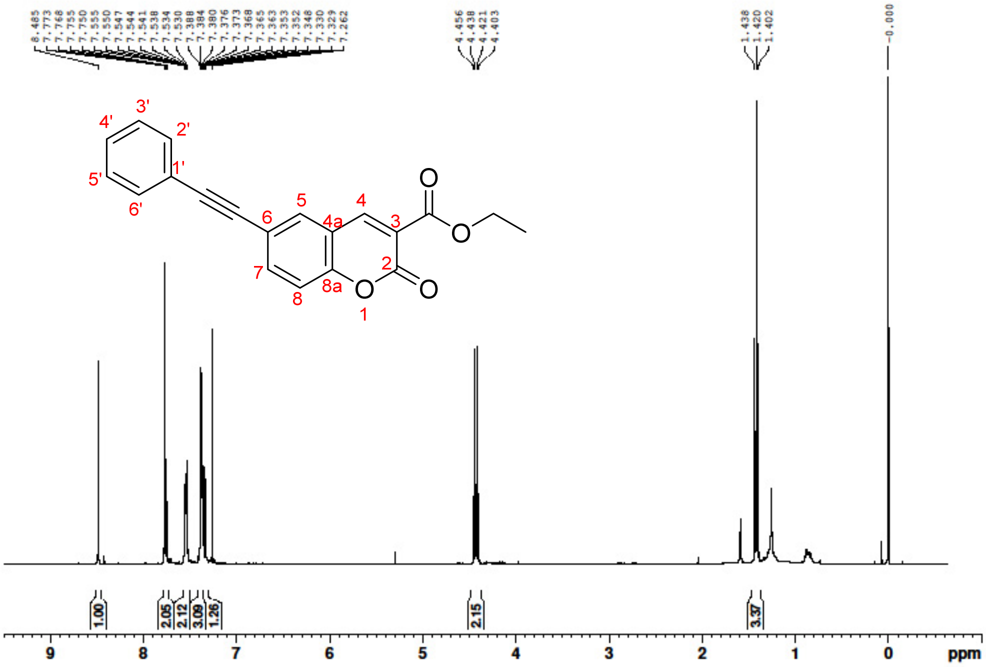


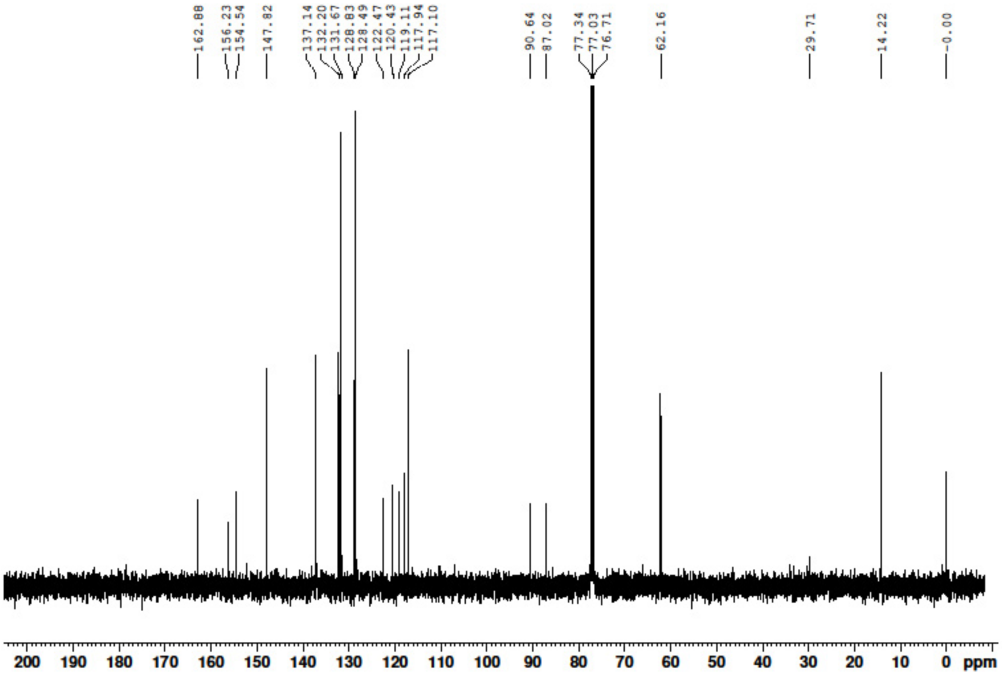

$^{1}$H, $^{13}$C and $^{31}$P NMR spectra of compound **12d**

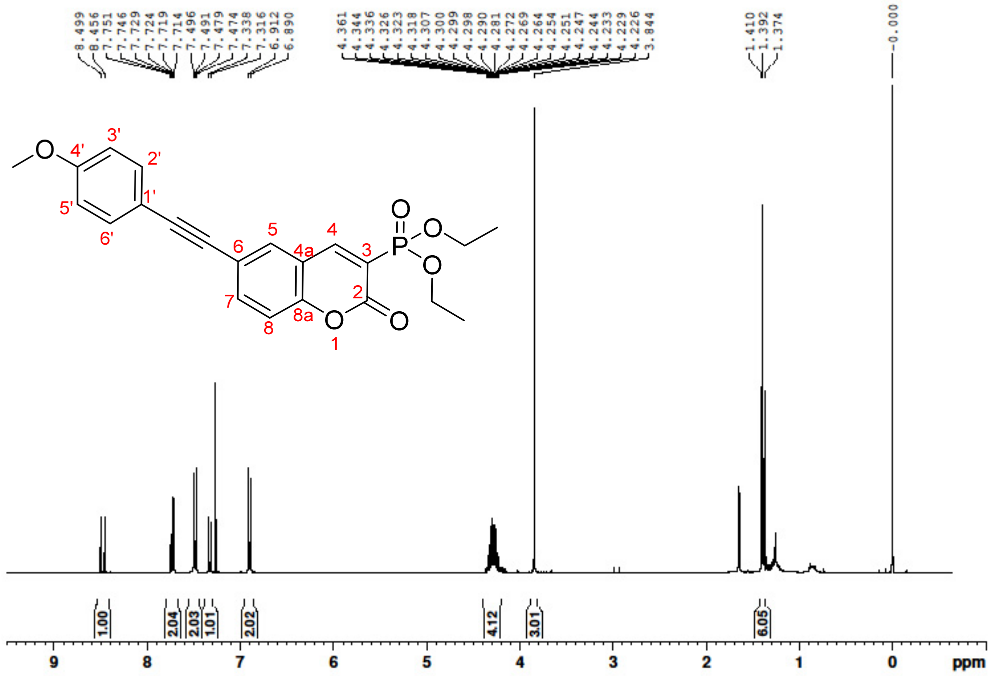


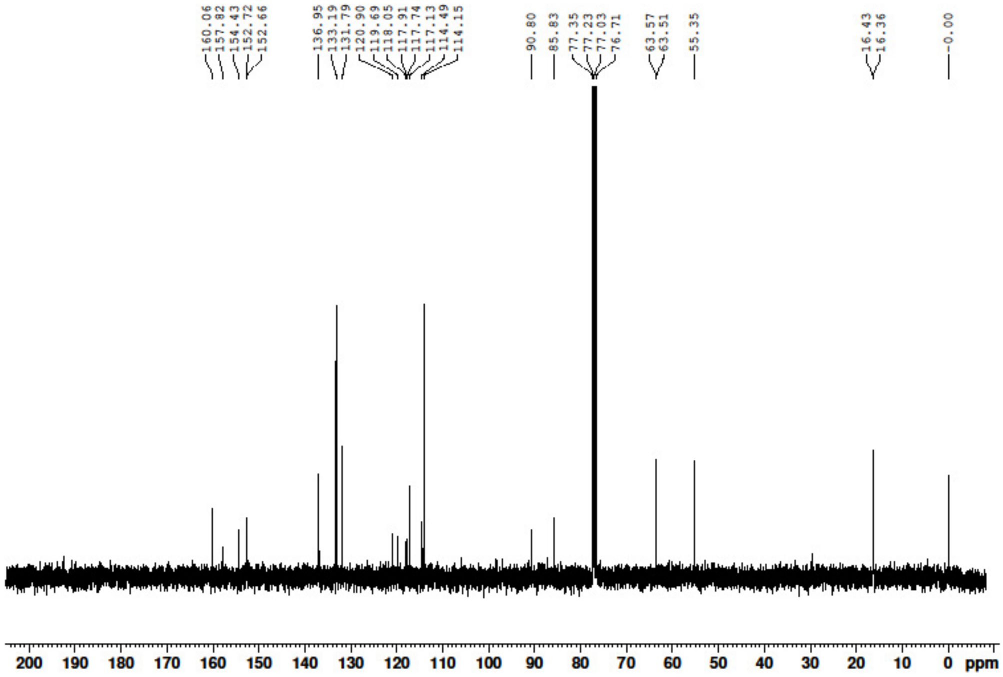

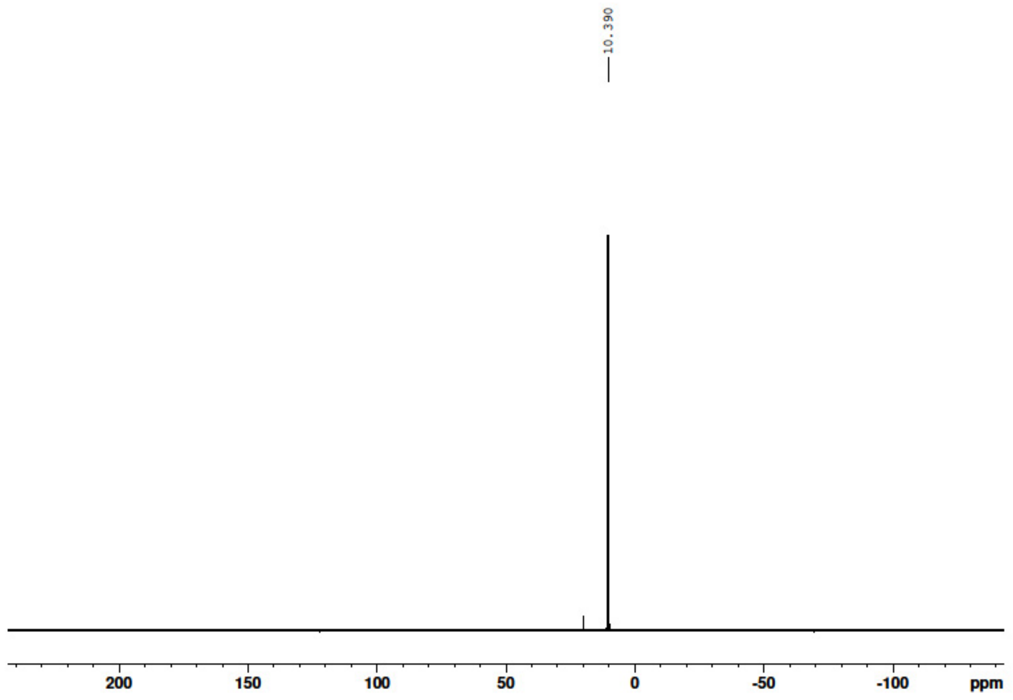
10.390
200
150
100
50
0
-50
-100
ppm
