# Supplementary material for: Efficient Synthesis of Fluorescent Coumarins and Phosphorous-Containing Coumarin-Type Heterocycles via Palladium Catalyzed Cross-Coupling Reactions
Source: Molecules. 2022 Nov 7;27(21):7649. doi: 10.3390/molecules27217649 (PMC9654183; doi:10.3390/molecules27217649)
Supplement: Supplementary file 1 [file molecules-27-07649-s001.zip › X-ray_revised.pdf]

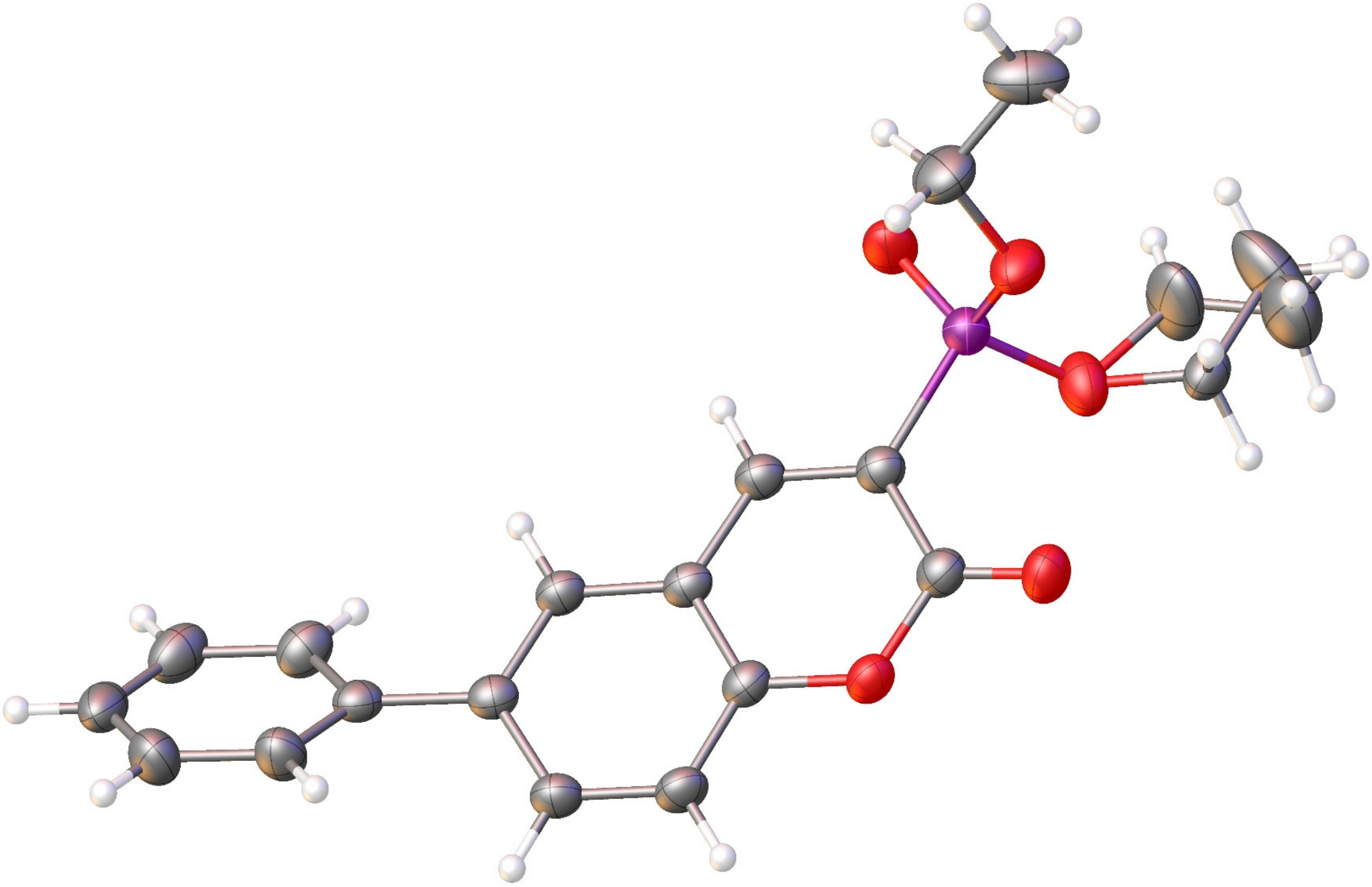

Diethyl (2-oxo-6-phenyl-2H-chromen-3-yl)phosphonate

Table S1** Crystal parameters of **3a

Crystal parameters		Crystal parameters		Crystal parameters	
**Empirical formula**	$C_{19}H_{19}O_5P$	**c/Å**	14.9033(9)	**F(000)**	752.9
**Formula weight**	358.31	**α/°**	90	**Crystal size/mm^3**	0.28 × 0.24 × 0.22
**Temperature/K**	133.00	**β/°**	90.726(2)	**Radiation**	MoKα (λ = 0.71073)
**Crystal system**	monoclinic	**γ/°**	90	**Goodness of fit on F^2**	1.026
**Space group**	P 2_1/n	**Volume/Å^3**	1736.20(19)	**Data/restraints/ parameters**	4155/44/248
**a/Å**	13.6869(9)	**Z**	4	**final R indices (I > 2σ(I))**	R_1 = 0.0390, wR_2 = 0.1052
**b/Å**	8.5123(5)	**μ/mm^{-1}**	0.185	**R indices (all data)**	R_1 = 0.0438, wR_2 = 0.1092

***Table S2** Fractional Atomic Coordinates (×10^4) and Equivalent Isotropic Displacement Parameters (Å^2×10^3) for **3a**. U_{eq} is defined as 1/3 of of the trace of the orthogonalised U_{IJ} tensor*

Atom	x	y	z	U(eq)
P1	7040.7(3)	4040.7(4)	5145.4(3)	32.53(12)
O1	6337.5(7)	-158.8(11)	6294.7(7)	32.2(2)
O2	7770.0(7)	936.5(13)	6043.1(8)	41.1(3)
O3	6368.9(8)	5178.5(13)	4736.3(8)	45.5(3)
O4	7782.9(8)	3289.6(14)	4491.4(7)	42.6(3)
O5	7705.0(8)	4732.7(13)	5920.3(8)	43.0(3)
C1	6897.5(10)	1072.6(15)	5978.8(9)	30.7(3)
C2	6373.0(10)	2421.2(15)	5598.2(9)	29.0(3)
C3	5387.6(10)	2468.9(15)	5597.9(9)	29.0(3)
C4	4829.1(10)	1214.7(14)	5981.3(8)	27.0(3)
C5	5332.7(10)	-81.6(15)	6325.6(8)	27.8(3)
C6	4841.7(10)	-1321.8(15)	6716.8(9)	32.2(3)
C7	3835.5(10)	-1253.3(15)	6777.8(9)	31.0(3)
C8	3299.0(10)	44.1(14)	6452.5(8)	27.6(3)
C9	3808.5(9)	1256.6(15)	6047.9(8)	27.8(3)
C10	2223.4(10)	142.8(14)	6550.3(9)	29.1(3)
C11	1760.7(11)	-452.7(17)	7303.8(11)	37.8(3)
C12	755.1(12)	-345.2(19)	7391.3(13)	45.6(4)
C13	191.5(11)	352.5(18)	6727.9(12)	42.6(4)
C14	638.8(12)	959.7(19)	5983.5(12)	44.2(4)
C15	1646.8(11)	865.9(17)	5891.8(10)	37.8(3)
C16	7330.4(14)	5918(2)	6527.6(13)	51.3(4)
C17	8129.3(16)	7003(2)	6784.5(13)	57.3(5)
C18A	8812.3(13)	3272(2)	4551.8(13)	34.4(5)
C19A	9235(4)	4457(7)	3944(4)	80.9(17)

C18B	8445(7)	4221(11)	3967(7)	70(3)
C19B	9407(14)	3960(20)	4055(16)	72(4)

Table S3 *Anisotropic Displacement Parameters (Å^2×10^3) for* ***3a.*** *The Anisotropic displacement factor exponent takes the form: $-2\pi^2[h^2a^{*2}U_{11}+2hka^*b^*U_{12}+\ldots]$.*

Atom	U_{11}	U_{22}	U_{33}	U_{12}	U_{13}	U_{23}
P1	30.11(19)	27.27(19)	40.3(2)	0.67(12)	3.36(14)	2.93(13)
O1	30.9(5)	28.1(5)	37.5(5)	6.2(4)	1.9(4)	5.3(4)
O2	31.5(5)	40.4(6)	51.5(6)	6.8(4)	3.0(4)	6.8(5)
O3	38.2(6)	34.8(6)	63.5(7)	2.1(4)	3.7(5)	19.1(5)
O4	34.5(5)	50.1(6)	43.4(6)	-5.0(5)	8.2(4)	-6.3(5)
O5	38.4(6)	36.2(6)	54.3(7)	2.4(4)	-0.1(5)	-13.9(5)
C1	33.1(7)	28.7(6)	30.4(6)	3.7(5)	3.5(5)	0.7(5)
C2	32.4(6)	26.2(6)	28.4(6)	2.2(5)	1.7(5)	1.4(5)
C3	33.9(6)	24.6(6)	28.4(6)	2.6(5)	0.6(5)	2.2(5)
C4	32.9(6)	23.6(6)	24.5(6)	3.4(5)	1.1(5)	0.9(4)
C5	30.8(6)	25.7(6)	26.7(6)	4.6(5)	0.9(5)	-1.2(5)
C6	36.7(7)	24.4(6)	35.4(7)	5.4(5)	0.0(5)	4.8(5)
C7	36.0(7)	23.9(6)	33.2(7)	0.5(5)	0.3(5)	3.3(5)
C8	32.8(6)	23.9(6)	26.1(6)	0.8(5)	-1.1(5)	-1.4(4)
C9	32.4(6)	23.9(6)	27.0(6)	3.7(5)	-0.8(5)	1.7(4)
C10	32.5(6)	21.6(6)	33.0(7)	0.0(5)	-1.9(5)	-2.3(5)
C11	34.1(7)	34.4(7)	45.0(8)	-1.2(6)	0.6(6)	10.0(6)
C12	36.5(8)	39.2(8)	61.3(10)	-3.5(6)	8.2(7)	8.0(7)
C13	30.1(7)	30.9(7)	66.6(10)	1.3(5)	-1.9(7)	-10.0(7)
C14	41.4(8)	41.2(8)	49.8(9)	11.2(6)	-11.3(7)	-6.1(7)
C15	40.0(8)	37.6(7)	35.8(7)	7.5(6)	-3.8(6)	-0.3(6)
C16	57.1(10)	40.3(8)	56.7(10)	5.1(7)	10.4(8)	-12.8(7)
C17	89.6(14)	35.2(8)	46.9(9)	-2.3(8)	-12.9(9)	-1.8(7)
C18A	28.6(9)	33.2(10)	41.3(10)	5.5(7)	-1.4(7)	-5.9(7)

1. *Experimental*

The data set was collected using a Bruker D8 Venture diffractometer with a microfocus sealed tube and a Photon II detector. Monochromated $Mo_{K\alpha}$ radiation (λ = 0.71073 Å) was used. Data were collected at 133(2) K and corrected for absorption effects using the multi-scan method. The structure was solved by direct methods using SHELXT [1] and was refined by full matrix least squares calculations on F^2 (SHELXL2018 [2]) in the graphical user interface Shelxle [3].

[1] Sheldrick, G. M. (2015). Acta Cryst. A71, 3-8.
[2] Sheldrick, G. M. (2015). Acta Cryst. C71, 3-8.
[3] Hübschle, C. B., Sheldrick, G. M., Dittrich, B. (2011). J. Appl. Crystallogr., 44, 12811284.

2. Refinement

All non H-atoms were located in the electron density maps and refined anisotropically. C-bound H atoms were placed in positions of optimized geometry and treated as riding atoms. Their isotropic displacement parameters were coupled to the corresponding carrier atoms by a factor of 1.2 (CH, CH_2) or 1.5 (CH_3). Disorder: The ethoxy-group on O5 was split over two positions. Its occupancy factors refined to 0.775 for the major compound.

3. Crystal Data

Crystal Data for $C_{19}H_{19}O_5P$ (M = 358.333g/mol): monoclinic, space group $P2_1/n$ (no. 14), a = 13.6869(9) Å, b = 8.5123(5) Å, c = 14.9033(9) Å, α = 90°, β = 90.726(2)°, γ = 90° V = 1736.20(19) Å3, Z = 4, T = 133.00 K, μ(MoKα) = 0.185 mm^{-1}, Dcalc = 1.371 g/cm3, 26402 reflections measured (4.06° ≤ 2Θ ≤ 55.84°), 4155 unique (R_{int} = 0.0591, R_{sigma} = 0.0405) which were used in all calculations. The final R_1 was 0.0393 (I > 2σ(I)) and wR_2 was 0.1111 (all data).
